# “Small” Intestinal Immunopathology Plays a “Big” Role in Lethal Cytokine Release Syndrome, and Its Modulation by Interferon-γ, IL-17A, and a Janus Kinase Inhibitor

**DOI:** 10.3389/fimmu.2020.01311

**Published:** 2020-06-26

**Authors:** Shiv D. Kale, Brittney N. Mehrkens, Molly M. Stegman, Bridget Kastelberg, Henry Carnes, Rachel J. McNeill, Amy Rizzo, Saikumar V. Karyala, Sheryl Coutermarsh-Ott, Jackie A. Fretz, Ying Sun, Jonathan L. Koff, Govindarajan Rajagopalan

**Affiliations:** ^1^Fralin Life Sciences Institute, Virginia Tech, Blacksburg, VA, United States; ^2^The Discipline of Microbiology and Immunology, Edward via College of Osteopathic Medicine, Blacksburg, VA, United States; ^3^College of Sciences, Virginia Tech, Blacksburg, VA, United States; ^4^Research and Graduate Studies, Virginia-Maryland College of Veterinary Medicine, Virginia Tech, Blacksburg, VA, United States; ^5^Office of the University Veterinarian, Virginia Tech, Blacksburg, VA, United States; ^6^Genomics Sequencing Center, Fralin Life Sciences Institute, Virginia Tech, Blacksburg, VA, United States; ^7^Department of Biomedical Sciences and Pathobiology, Virginia-Maryland College of Veterinary Medicine, Virginia Tech, Blacksburg, VA, United States; ^8^Histology and Histomorphometry Laboratory, Department of Orthopedics and Rehabilitation, Yale School of Medicine, New Haven, CT, United States; ^9^Division of Pulmonary Critical Care and Sleep Medicine, Department of Medicine, Yale School of Medicine, New Haven, CT, United States

**Keywords:** cytokine release syndrome, superantigens, HLA class II transgenic mice, multiple organ dysfucntion, JAK inhibitor

## Abstract

Chimeric antigen receptor T cell (CART) therapy, administration of certain T cell-agonistic antibodies, immune check point inhibitors, coronavirus disease 2019 (COVID-19) caused by severe acute respiratory syndrome–coronavirus 2 (SARS-CoV-2) and Toxic shock syndrome (TSS) caused by streptococcal as well as staphylococcal superantigens share one common complication, that is T cell-driven cytokine release syndrome (CRS) accompanied by multiple organ dysfunction (MOD). It is not understood whether the failure of a particular organ contributes more significantly to the severity of CRS. Also not known is whether a specific cytokine or signaling pathway plays a more pathogenic role in precipitating MOD compared to others. As a result, there is no specific treatment available to date for CRS, and it is managed only symptomatically to support the deteriorating organ functions and maintain the blood pressure. Therefore, we used the superantigen-induced CRS model in HLA-DR3 transgenic mice, that closely mimics human CRS, to delineate the immunopathogenesis of CRS as well as to validate a novel treatment for CRS. Using this model, we demonstrate that (i) CRS is characterized by a rapid rise in systemic levels of several Th1/Th2/Th17/Th22 type cytokines within a few hours, followed by a quick decline. (ii) Even though multiple organs are affected, small intestinal immunopathology is the major contributor to mortality in CRS. (iii) IFN-γ deficiency significantly protected from lethal CRS by attenuating small bowel pathology, whereas IL-17A deficiency significantly increased mortality by augmenting small bowel pathology. (iv) RNA sequencing of small intestinal tissues indicated that IFN-γ-STAT1-driven inflammatory pathways combined with enhanced expression of pro-apoptotic molecules as well as extracellular matrix degradation contributed to small bowel pathology in CRS. These pathways were further enhanced by IL-17A deficiency and significantly down-regulated in mice lacking IFN-γ. (v) Ruxolitinib, a selective JAK-1/2 inhibitor, attenuated SAg-induced T cell activation, cytokine production, and small bowel pathology, thereby completely protecting from lethal CRS in both WT and IL-17A deficient HLA-DR3 mice. Overall, IFN-γ-JAK-STAT-driven pathways contribute to lethal small intestinal immunopathology in T cell-driven CRS.

## Introduction

With a surge in clinical use of chimeric antigen receptor T cells (CART), T cell agonistic antibodies and immune check point inhibitors, there is a concomitant rise in the incidence of cytokine release syndrome (CRS), a T cell-mediated systemic disease (1). In these above therapeutic modalities, T cells get robustly activated by tumor antigens, agonistic antibodies, or by removal of inhibitory signals and produce large amounts of T cell-derived cytokines such as IFN-γ, IL-17, and TNF-α. These cytokines in turn activate macrophages and other immune cells to produce several additional cytokines. Activated T cells in conjunction with these cytokines cause extensive immunopathology to multiple vital organs, ultimately leading to their failure, which is clinically defined as multiple organ dysfunction or MOD (1, 2). MOD is often accompanied by hypotension and could become irreversible, thus making CRS a life-threatening condition (1, 3–6). Mortality associated with the ongoing global pandemic coronavirus disease 2019 (COVID-19), caused by the severe acute respiratory syndrome–coronavirus 2 (SARS-CoV-2), is also believed to result from serious CRS (7, 8).

There is no specific treatment available to date for CRS, and it is managed only symptomatically to support the deteriorating organ functions and maintain the blood pressure (2). As cytokine(s) produced by activated T cells are primarily responsible for mediating MOD, identifying the key pathogenic cytokine that contributes to MOD in CRS, and delineating the underlying molecular pathways could enable more precise treatment of CRS as in other inflammatory diseases (9). Also, identifying whether the failure of any one particular organ contributes more significantly to the severity of CRS than the other organs can also help in more targeted and efficient treatment of CRS. Hence, we used the superantigen-induced CRS model in HLA-DR3 transgenic mice, that closely mimics human CRS, to delineate the immunopathogenesis of CRS as well as to validate a novel treatment for CRS.

Superantigens (SAgs) are a family of potent exotoxins produced by *Staphylococcus aureus* and *Streptococcus pyogenes*. SAgs are robust activators of T cells (10, 11). SAgs bind directly to MHC class II molecules outside of the peptide-binding groove without undergoing any processing. Subsequently, they interact directly with selected T cell receptor (TCR) variable region (Vβ or Vα) families, and CD28 on T cells, crosslink the TCRs and CD28, thereby causing a robust polyclonal activation of 40–60% of all CD4^+^ and CD8^+^ αβ TCR^+^ adaptive T cells (12). T cells activated by SAgs rapidly produce abundant amounts of pro-inflammatory cytokines resulting in CRS. SAgs can also activate innate T cells such as natural killer T (NKT) cells and mucosal associated invariant T cells (MAITs) to produce cytokines by a similar mechanism, thus heightening the severity of CRS.

Bacterial SAgs bind more efficiently to human MHC (called HLA) class II molecules than to murine MHC class II. We and others have shown that acute CRS with life-threatening MOD, analogous to human CRS, can be readily induced in HLA class II transgenic mice with purified SAgs alone in the absence of infection (13–16). SAg-induced CRS in HLA class II transgenic mice is broadly applicable to T cell-driven CRS such as treatment with CART cells, T cell agonistic antibodies, immune check point inhibitors, haploidentical hematopoietic stem cell transplantation, and others. Therefore, we used the purified SAg to induce CRS in HLA-DR3 transgenic mice to delineate the roles of IFN-γ and IL-17A, the signature pro-inflammatory cytokines produced by activated CD4^+^ and CD8^+^ T cells, in the immunopathogenesis of CRS and validated a JAK-1/2 inhibitor, ruxolitinib for the treatment of CRS.

## Materials and Methods

### Mice

AE°.HLA-DR3 transgenic mice expressing HLA-DRA1^*^0101 and HLA-DRB1^*^0301 transgenes on the complete mouse MHC-II–deficient background (AE°), HLA-DR3.Nur-77 GFP transgenic mice expressing green fluorescent protein (GFP) under the control of Nur-77 promoter and AE°.HLA-DR3.IFN-γ^−/−^ mice are described elsewhere (14, 15, 17–19). AE°.HLA-DR3.IL-17A^−/−^ mice were produced by crossing IL-17A-deficient mice obtained from Dr. Yoichiro Iwakura, Tokyo University of Science (20), with AE°.HLA-DR3 mice for several generations. Absence of endogenous mouse MHC class II molecules, absence of *Ifng*, and *Il17a* genes and the presence of various transgenes were confirmed by PCR. Mice of either sex, spanning 8–14-weeks of age were used in the experiments. All animal experiments were approved by the Virginia Tech Institutional Animal Care and Use Committee and the Office of Laboratory Animal Welfare assurance number is A-3208-01.

### Reagents and Antibodies

Staphylococcal enterotoxin B, in its highly purified, endotoxin-reduced form was purchased from Toxin Technology Inc. (Sarasota, FL). A stock solution of 1 mg/ml in phosphate buffered saline (PBS) was stored frozen in aliquots at −20°C. Ruxolitinib (Selleckem, Houston, TX) was prepared as per manufacturer's instruction. Briefly, ruxolitinib was dissolved in pure dimethyl sulfoxide (DMSO) to make 100 mg/ml stock solution, aliquoted and stored frozen in aliquots at −20°C. For oral gavage, PEG300, and distilled water were added to the stock solution as suggested by the manufacturer. The following antibodies from BioLegend (San Diego, CA) were used for flow cytometry. Anti-CD4 (clone GK1.5), anti-CD8 (clone 53-6.7), TCR Vβ6 (clone RR 4-7), and TCR Vβ8 (KJ16-133.18 or MR5-2). anti-CD25 (clone PC61) and anti-CD69 (clone H1.2F3).

### Induction of SAg-Induced CRS and Administration of Compounds

Mice were challenged with 50 μg of SEB in 200 μl of PBS, administered via intraperitoneal injection. Mice were euthanized at 6 h or at indicated time points and blood collected by cardiac puncture. Sera were then used for cytokine analyses. In preliminary studies, ruxolitinib at 100 mg/kg was found to be toxic. In all subsequent experiments, ruxolitinib was used at a dose of 50 mg/kg. When ruxolitinib was used prophylactically, animals were weighed, and gavaged with ruxolitinib once at 9 AM. and once at 4 PM. The next day, mice were challenged with SEB at 9 AM. Twice daily oral gavage with ruxolitinib continued unless stated otherwise. In experiments were ruxolitinib was used simultaneously with SEB, animals were weighed and challenged with SEB. Immediately afterwards, mice were gavaged with ruxolitinib and again at 4 PM. Twice daily oral gavage with ruxolitinib continued for 3 more days after SEB challenge. Mice were monitored frequently for external symptoms of severe distress as recommended by the Institutional Animal Care and Use Committee. Moribund animals were removed from the study as per IACUC recommendation. Animals were weighed on every other day.

### *In vitro* Splenocyte Cultures

Spleens from HLA-DR3 or HLA-DR3.GFP mice were collected aseptically immediately after euthanasia. Mononuclear cells were prepared by pressing the spleens through a nylon sieve (100 μM sieve size, Corning) with a plunger of a plastic syringe as per standard techniques. Red blood cells were lysed using ammonium chloride, washed, counted, and resuspended in complete RPMI medium containing serum. Cells were cultured with medium alone or with indicated concentrations of SEB in the presence of ruxolitinib or vehicle for 48 h in 24-well-tissue culture plates. Ruxolitinib or vehicle were added immediately after SEB. After the incubation period, the cells were collected, spun, and the supernatants were used to measure the concentrations of cytokines. For determining the expression of activation markers, the cell pellets were washed once and stained with indicated antibodies for flow cytometry. Expression of GFP and the activation markers CD25 and CD69 on CD4^+^- and CD8^+^-gated T cells was determined.

To determine the effect of ruxolitinib on SEB-induced cell proliferation, splenic total T lymphocytes were isolated from HLA-DR3 transgenic mice using a pan-T cell negative selection kit (Miltenyi Biotec, Auburn, CA) as per manufacturer's guidelines. Purified T cells were labeled with CFSE (0.5 μM) following manufacturer's protocol (CellTrace™ CFSE Cell Proliferation Kit, Thermo Fisher Scientific, Waltham, MA). The non-T cell fraction was eluted from the column and used as antigen presenting cells (APCs). CFSE-labeled T cells were cultured with APCs in the presence of medium alone or with indicated concentrations of SEB. Ruxolitinib or vehicle were added to the cultures and incubated for 48 h. After the incubation period, the cells were washed, stained with antibodies, and the extent of T cell proliferation was determined by flow cytometry by measuring reduction in CFSE intensity over time.

### Histopathological Analyses and Terminal Deoxynucleotidyl Transferase dUTP Nick End Labeling (TUNEL) Staining of Intestinal Tissue

Mice were euthanized and necropsied 6, 24, and 48 h post-PBS or -SEB exposure. Samples of liver, kidney, lung, and small intestine were placed into 10% neutral buffered formalin for at least 24 h prior to submission to the diagnostic lab at Virginia-Maryland College of Veterinary Medicine for routine processing. Samples were routinely embedded in paraffin, sectioned at 5 μm thickness and stained with hematoxylin and eosin (H&E). Tissues were evaluated and graded for inflammation, and cell death by a board-certified veterinary pathologist (SCO). Sections of the lungs were graded according to the presence and severity of perivascular inflammation (0–3), peribronchial, and peribronchiolar inflammation (0–3), and interstitial inflammation (0–3). Sections of intestines were graded according to the presence and severity of 3 histopathologic parameters: inflammatory infiltrates (0–4), epithelial damage (0–5), and disruption of mucosal architecture (0–3). Sections of liver were graded according to the presence and severity of inflammation (0–4), extramedullary hematopoiesis (0–4), and cell death (0–4). TUNEL staining of formalin-fixed paraffin-embedded small intestinal tissue samples were done using the TdT *in situ* Apoptosis Detection Kit – DAB (Cat # 4810-30-K, R&D Systems, Inc., MN USA) at Histology and Histomorphometry Laboratory (Yale School of Medicine, New Haven, CT) as per recommended protocol.

### Cytokine Analyses

The cytokine/chemokine concentrations in individual serum samples or culture supernatants were determined using a multiplex bead assay (Invitrogen™ Cytokine & Chemokine Convenience 26-Plex Mouse ProcartaPlex™ Panel 1, Thermo Fisher Scientific, Waltham, MA) per the manufacturer's protocol and using Luminex MagPix hardware and software (Life Technologies Corporation, ThermoFisher Scientific, Waltham, MA). For serum cytokine analysis, mice were sacrificed at indicated time points. At the time of sacrifice, blood was collected in serum separation tubes (BD Biosciences, Franklin Lakes, NJ), sera were separated by centrifugation and stored frozen at −80°C in aliquots. For analyzing culture supernatants, after indicated time points, the supernatants were harvested, centrifuged, and the cell-free supernatants were used in the assay.

### Flow Cytometry

Mice challenged with 50 μg of SEB and treated with either ruxolitinib or vehicle were euthanized at 48 h. Splenic mononuclear cells were prepared as described earlier, resuspended in PBS, and processed for flow cytometry as per standard procedure using indicated antibodies. When cultured cells were used, cells were harvested from plates, washed once, stained as per standard procedures, and used for flow cytometry. Flow data was analyzed using the FlowJo software version 10. Mononuclear cells were further gated based on CD4 and CD8 expression and expression of CD25, CD69, and GFP were studied on these gated populations.

### RNA Sequencing and Bioinformatics Analysis

Age-matched DR3.WT, DR3.IFN-γ°, and DR3.IL-17° mice of either sex were challenged intraperitoneally with 50 μg of SEB. Experimental animals were euthanized at 6 or 24 h, small intestines were removed, and small intestinal tissues (from jejunum) were collected and stored in RNAlater until processing. RNA from tissue samples was extracted using Trizol (Invitrogen) by homogenizing the samples in using Precellys Evolution tissue homogenizer (Bertin Insteruments, Rockville, MD, USA). RNA was further purified using Qiagen columns following manufacturers' instructions. Total RNA with RIN ≥8.0, was converted into a strand-specific library using Illumina's TruSeq Stranded mRNA HT Sample Prep Kit (Illumina, RS-122-2103), for subsequent cluster generation and sequencing on Illumina's NextSeq. The library was enriched by 13 cycles of PCR, validated using Agilent TapeStation, and quantitated by qPCR. Individually indexed cDNA libraries were pooled and sequenced on NextSeq.

Generated sequences were trimmed for residual adaptors and poor quality bases (sliding window 4, phred score 25) and length (>36) using Trimmomatic-0.35 (21). Non-paired reads were also filtered out. Filtered paired-end reads were aligned to the mouse reference genome (GRCm38.94) using HiSAT2. Transcript assembly and quantification of RNA-Seq was done via Stringtie, with output specific for DESeq2 (22–24). DESeq2 pipeline was conducted to determine normalized gene counts and differential expression. Differential expression was conducted in a pair-wise manner between genotypes or treatments and further filtered using a threshold criterion of log2fold Δ >1 p_adj_ < 0.05 (Supplementary Figure 1). Raw data, raw and normalized count files, list of differential expressed genes can be accessed in the gene expression omnibus (GSE130125). Bioinformatics analysis was done through via custom scripts in R and python (available from Kale Lab), and cytoscape using the reactome plugin (25, 26). Clustering analysis was done through Morpheus version for R (https://software.broadinstitute.org/morpheus).

### Real-Time qRT-PCR

Expression levels of representative immune-related genes were further confirmed by real-time quantitative RT-PCR. 96-well-arrays preloaded with primers for the genes of interest, two house-keeping genes (β-actin and GAPDH), reverse-transcription controls, positive PCR controls, and genomic DNA controls were custom ordered from Qiagen. Rest of the qRT-PCR was performed using RT^2^ First Strand Kit and SYBR Green Mastermix (Qiagen) using QuantStudio™ 3 real-time PCR machine (Thermo Fisher Scientific) as per manufacturer's protocols. Melt curve data was exported to Microsoft Excel, and the mean relative expression of the individual gene was calculated using ΔΔCt method.

### Statistical Analysis

Survival curves were plotted using Prism 8 software, version 8.4.2 (GraphPad Software, San Diego, CA) and their statistical significances were determined using the log rank test, the Mantel-Cox method. Unless specifically indicated, unpaired Mann-Whitney test was used to compare 2 groups. All charts, unless indicated, were also prepared using the same software. Statistical tests used in RNA sequencing is described under RNA sequencing section.

## Results

### IL-17A Deficiency Enhanced, Whereas IFN-γ Deficiency Protected From Lethal CRS Induced by SAg

**Figure 1:**
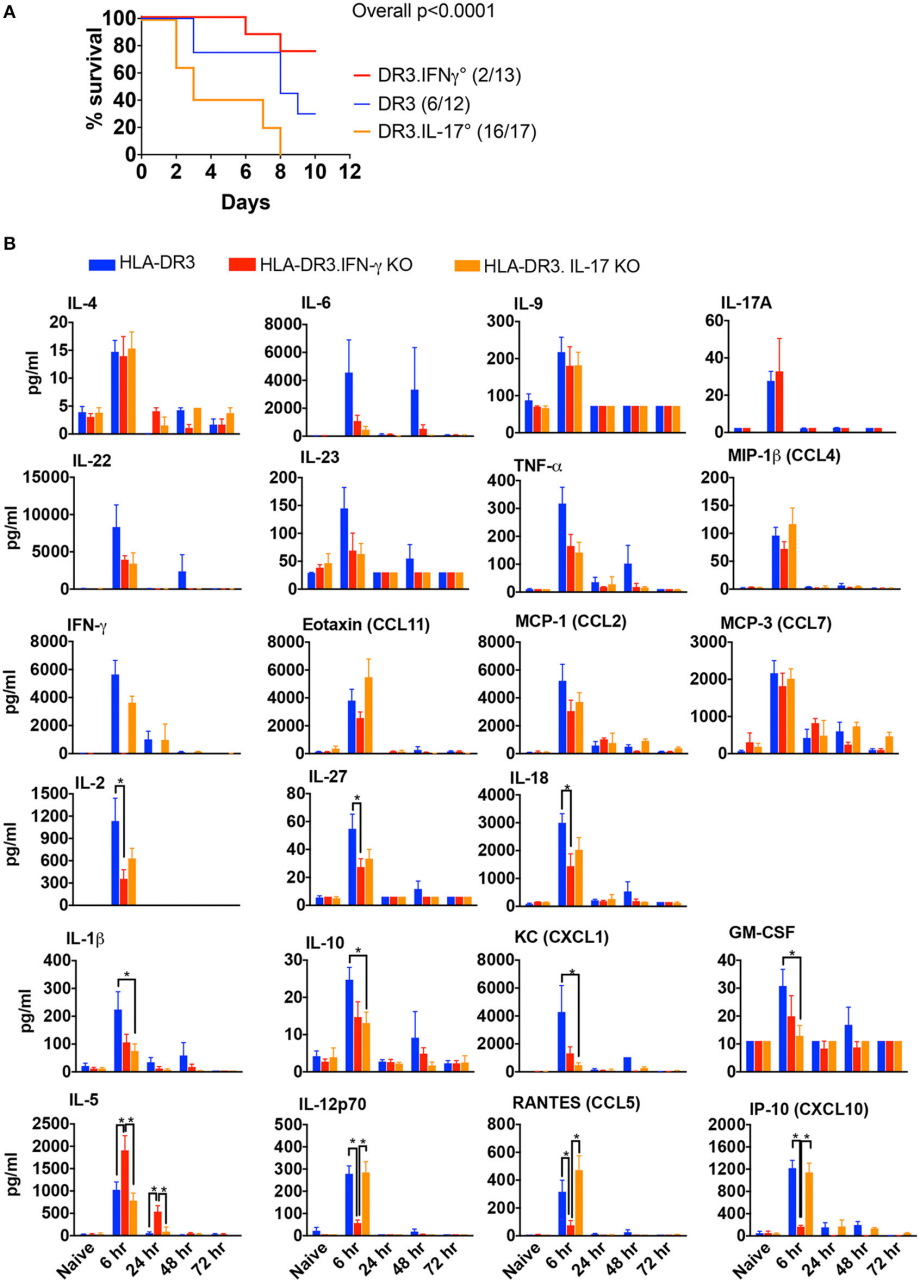
SAg-induced, T cell driven-CRS in HLA-DR3 mice and the impact of IFN-γ and IL-17-deficiency. **(A)** Mortality HLA-DR3.WT, DR3.IFN-γ°, and DR3.IL-17° mice were challenged with 50 μg of SEB in 200 μl of PBS by intraperitoneal route. Mice were monitored frequently as per IACUC recommendations. Moribund animals and other animals that met the criteria for humane endpoints were removed from the study and euthanized. Survival curves were plotted using graphpad prism. Figures in parenthesis indicate the number of animals that succumbed over total number of mice used. **(B)** Cytokine signature mice challenged as in **(A)** were euthanized at indicated time points and blood collected by cardiac puncture. Concentrations of a panel of cytokines and chemokines in individual serum samples were determined using multiplex bead assay. Each bar represents mean ± SE from 3 to 10 mice. *p < 0.05.

HLA-DR3 mice upon challenge with SEB develop classical CRS along with MOD and succumb to the disease over a 3–7 day period (14). Thus, HLA-DR3 mice were ideal tool to delineate the roles of IFN-γ and IL-17A in SAg-induced CRS. While wild-type HLA-DR3 mice (DR3.WT) succumbed to CRS, DR3.IFN-γ° mice were significantly protected, replicating our previous findings (14). However, DR3.IL-17° mice were highly susceptible to CRS (Figure 1A), indicating that IFN-γ and IL-17A play opposing roles in CRS; IL-17A is protective, whereas IFN-γ is pathogenic.

### Effect of IFN-γ and IL-17A-Deficiency on the *in vivo* Immune Responses to SEB

Given the contrasting roles for IL-17A and IFN-γ in the outcome of CRS, we next studied the characteristics of the systemic cytokine/chemokine storm induced by SEB in DR3.WT mice and HLA-DR3 mice lacking these cytokines. Several Th1-, Th2-, Th17-, Th22-type cytokines as well as chemokines were rapidly elevated in DR3.WT mice during CRS as previously shown (15). Concentrations of serum cytokines ad chemokines generally peaked by 6 h and returned to base line by 72 h (Figure 1B). In DR3.IFN-γ° mice, serum levels of IL-12p70, IL-18, IL-27, and IL-2, and chemokines (CXCL10, CCL9) were lower, while IL-5, a key Th2-type cytokine, was significantly higher. IL-17A was only slightly elevated in DR3.IFN-γ° mice (*p* = NS). We expected that the serum levels of many pro-inflammatory cytokines and chemokines will be higher in DR3.IL-17° mice because they had the highest mortality. On the contrary, many of the inflammatory mediators were lower compared to DR3.WT mice, some of these differences being significant (IL-1β, IL-10, CXCL1, and GM-CSF), while others were not (IL-6, IFN-γ, and TNF-α) (Figure 1B).

### Organ Pathology in SAg-CRS and Its Modulation by IFN-γ and IL-17A

**Figure 2:**
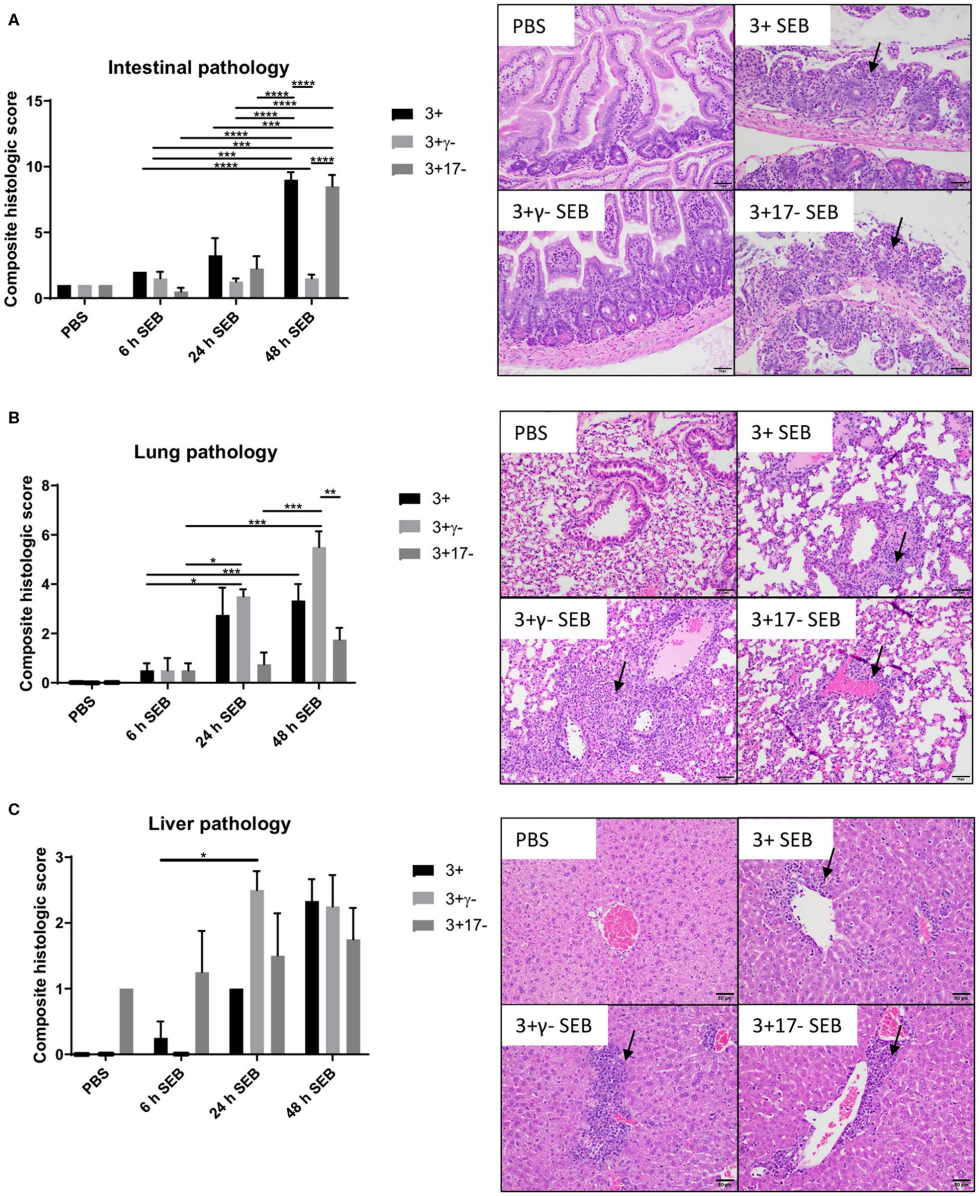
IFN-γ and IL-17A deficiency differentially modulate organ pathology associated with SAg-induced CRS. HLA-DR3.WT, DR3.IFN-γ°, and DR3.IL-17° mice were challenged with 50 μg of SEB in 200 μl of PBS by intraperitoneal route. Groups of mice were euthanized at indicated time points and organs collected in buffered formalin. Tissues were embedded in paraffin, processed, and stained with hematoxylin and eosin as per the standard procedure. Stained sections of **(A)** small intestine, **(B)** lung, and **(C)** liver were then graded by a board-certified veterinary pathologist (SCO) in a blinded fashion. Panels on the right shows representative images of H&E stained slides (from 48 h group) of certain fields taken at 20x magnification. Scores mean±SD from 4 to 8 mice per group. *p < 0.05, **p < 0.005, ***p < 0.0005, ****p < 0.0001.

Multi-organ inflammation resembling MOD seen in human sepsis/septic shock could be elicited during SAg-induced CRS in DR3.WT mice (15). Therefore, we next investigated if deficiency of IL-17A or IFN-γ changed the characteristics of organ pathology. In general, minimal histological changes were noticed in all the organs at 6 h in the three groups. However, at 24 h and more so at 48 h, both the DR3.WT and DR3.IL-17° mice had greater evidence of intestinal damage and thus higher composite intestinal pathology scores. Intestinal sections from these groups were characterized by severe distortion of intestinal architecture including severe blunting and/or loss of villi, erosion to ulceration of the mucosal epithelium, necrosis, and significant neutrophilic infiltration into the lamina propria (Figure 2, *p* < 0.0001). DR3.IFN-γ° mice, had pathology scores similar to those of the PBS groups with maintenance of normal tissue architecture and low numbers of loosely scattered lymphocytes and plasma cells throughout the lamina propria reflecting minimal intestinal damage in these animals (Figure 2).

With reference to lungs, minimal histological changes were seen at 6 h in the lungs from all three genotypes. However, DR3.IFN-γ° mice had the highest lung pathology scores at 24 and 48 h, while DR3.IL-17° mice had the lowest. At 24 and 48 h, DR3.IFN-γ° mice developed moderate amounts of neutrophilic inflammation surrounding airways and blood vessels as well as filling alveolar spaces and occasionally obscuring alveolar septa. These changes were not identified in the DR3.IL-17° mice. Neutrophilic inflammation was minimal and often limited to the connective tissues surrounding vessels and airways. This suggested a differential effect of IFN-γ and IL-17 on the lungs and small intestines in SAg-induced CRS. A loss of IFN-γ seemed to be protective to the small intestine, while in the lung, it was associated with more severe immunopathology. Sections of liver were also evaluated. While not significant, DR3.IFN-γ° mice overall tended to have larger inflammatory infiltrates (Figure 2).

Overall, deficiency of IFN-γ protected from small bowel pathology, whereas deficiency of IL-17 enhanced gut pathology. Thus, both IFN-γ and IL-17A targeted the small intestines during SAg-induced CRS. While IFN-γ played a pathogenic role, IL-17A played a protective role. Since DR3.IFN-γ° mice had the highest survival and lowest gut pathology, while DR3.IL-17° mice had the lowest survival and highest gut pathology, we can infer that IFN-γ-mediated gut failure likely plays a lethal role in SAg-induced CRS and that IL-17A protects from IFN-γ-mediated gut pathology.

### Mitigated Organ Pathology in IFN-γ Deficient Mice Was Not Due to Poor SEB-Driven T Cell Activation

**Figure 3:**
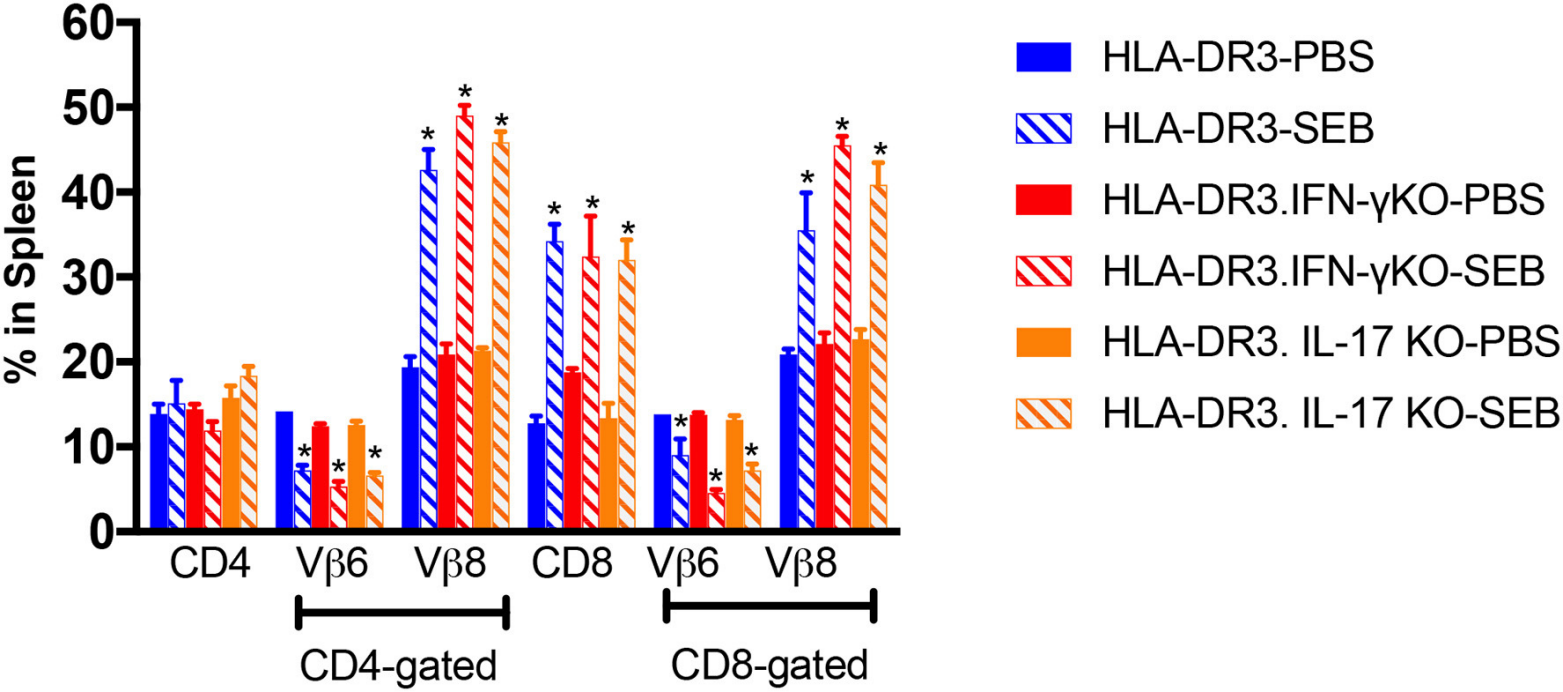
SAg-induced peripheral T cell expansion in IFN-γ and IL-17A-deficient mice. HLA-DR3.WT, DR3.IFN-γ°, and DR3.IL-17° were mice challenged with 50 μg of SEB in 200 μl of PBS by intraperitoneal route. Groups of mice were euthanized at 72 h post-SEB challenge and spleens were collected. Mononuclear cell suspensions of splenocytes were stained with fluorochrome-conjugated antibodies. Percentage of CD4^+^ and CD8^+^ T cells expressing TCR Vβ6 and Vβ8 were determined by flow cytometry. Each bar represents mean ± SE from 3 to 5 mice. *p < 0.05 compared to respective PBS control group.

We next studied the extent of SEB-induced expansion of T cells in these three strains of mice. As shown previously shown (15), administration of SEB caused an expansion of CD4^+^ and CD8^+^ T cells expressing TCR Vβ8 in DR3.WT mice (Figure 3). SEB-induced T cell expansion was only slightly higher in DR3.IL-17° mice (*p* = NS), in comparison to DR3.WT mice and was in highest in DR3.IFN-γ° mice (*p* = NS). Therefore, a heightened mortality in DR3.IL-17° mice cannot be simply explained by a more robust SEB-induced T cell proliferation, T cell expansion, and cytokine production. Similarly, lower mortality in DR3.IFN-γ° mice was not due to poor SEB-induced T cell activation and proliferation. This suggested that the molecular processes differentially induced by IL-17A and IFN-γ in various cells and tissues *in vivo*, particularly in the small intestines, likely determined the differential outcome.

### RNA Sequencing to Delineate the Molecular Pathways Underlying the Gut Pathology in SAg-Induced CRS and Its Modulation by IFN-γ and IL-17A

As the small bowel pathology was the distinguishing feature between mice that were protected and those succumbed to the disease, RNA sequencing was performed on total RNA isolated from the small intestinal tissue with two main objectives. (i) Describe the molecular pathways that were associated with small bowel immunopathology in CRS over time (6 and 24 h) in DR3.WT mice. (ii) Delineate the pathways that were likely modulated by IL-17-deficiency that lead to higher mortality in DR3.IL-17° mice and pathways that might have contributed to higher survival in DR3.IFN-γ° mice following experimental SAg-induced CRS.

**Figure 4:**
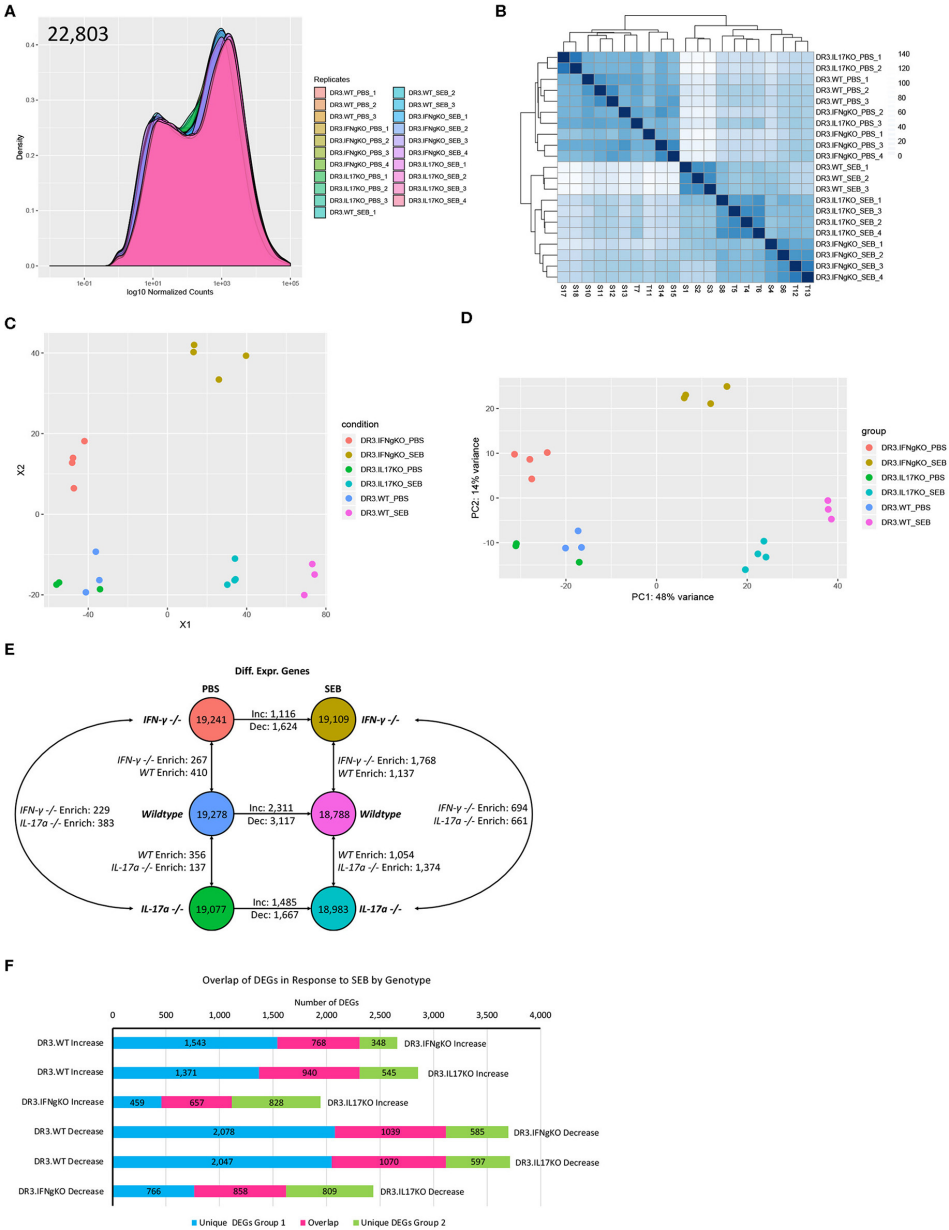
Analysis of global gene expression and differential expression in response to SAg-induced CRS. Genes with an expression value below 10 across all sample replicates were first excluded. **(A)** The distribution of gene expression across all sample replicates was determined for the filtered gene set. **(B)** Hierarchical clustering of samples based upon global filtered gene expression. **(C)** Multi-dimensional scaling and **(D)** principal coordinate analysis (PCoA) of global gene expression for each sample replicate. **(E)** Differential gene expression analysis for each experimental group. **(F)** Comparison of differentially expressed genes in response to SEB between genotypes. “Increase” indicates genes considered to be differentially expressed in an increasing manner (log_2_fold Δ >1, *p*_*adj*_ < 0.05) in response to SEB. “Negative” indicates genes considered to be differentially expressed in a decreasing manner in response to SEB (log_2_fold Δ >1, *p*_*adj*_ < 0.05).

PBS or SEB-challenged DR3.WT, DR3.IFN-γ°, and DR3.IL-17° mice were euthanized at 6-h post-SEB treatment. Experiments were designed to generate 30–40 million paired end reads that were then filtered to remove low-quality bases and reads, as well as unpaired mates prior to alignment and quantification (Supplementary Table 1). Genes with counts < 10 across all samples were removed and distribution of expression across samples was assessed (Figure 4A). This filtration resulted in 22,803 genes with expression above the threshold values. Hierarchical clustering of samples based on the filtered gene expression indicated all replicates grouped together with the exception of one DR3.IL-17° PBS sample, which clustered more closely to one of the DR3.IFN-γ° PBS sample replicates (Figure 4B). However, this deviation is considered to be marginal, given the short branch lengths between the PBS treatment groups. Hierarchical clustering of samples also showed separation primarily by treatment and then by genotype, suggesting that challenge with SEB induced the majority of genes expression changes.

We performed multi-dimensional scaling as well as principal coordinate analysis (PCoA) to gain insight into the similarity and dissimilarity of samples (Figures 4C,D). Samples within a treatment group clustered closely, with evident overlap between DR3.WT PBS and DR3. IL-17° PBS samples. Samples receiving either PBS or SEB treatment were separated across vector X1, while vector X2 highlighted the separation between DR3.IFN-γ° and the other two genotypes regardless of PBS or SEB treatment (Figure 4C). PCoA produced similar relationships and further indicated that 48% of observed overall variance (PC1) could be explained by SEB treatment, while 14% of the overall variance could be explained by gene expression differences associated primarily with DR3.IFN-γ° mice. We hypothesized that the top contributors to variance in PC2 might provide us insight into why DR3.IFN-γ° mice receiving SEB treatment may have enhanced survival and decreased intestinal epithelial damage. Analysis of PC3-8 did not elucidate any apparent association with SEB treatment and/or genotype (Supplementary Figure 2).

We then looked at differentially expressed genes between a subset of sample groups. Each sample group had between 18,788 and 19,278 genes considered to be expressed out of 22,803. Differential expression was then conducted via DESeq2. We further filtered the list of differentially expressed genes (DEGs) by making the log_2_fold change >1. This additional filtration reduced the number of DEGs by 38–70% (Supplementary Table 2, Supplementary Figure 3). Even with this enhanced stringency, we identified a range of 137–410 differentially expressed genes between the three genotypes suggesting that single gene knockout had significant effects on the basal gene expression even in PBS-challenged animals (Figure 4E).

Treatment of DR3.WT mice with SEB resulted in a total of 5,428 differentially expressed genes (2,311 increasing, 3,117 decreasing) in comparison to its PBS control. DR3.IFN-γ° mice treated with SEB resulted in 1,116 increasing and 1,624 decreasing genes in comparison to its PBS control, and DR3.IL-17° treated with SEB resulted in 1,485 increasing and 1,667 decreasing genes in comparison to its corresponding PBS control. We then sought to determine if each genotype was responding in a similar or divergent manner when treated with SEB (Figure 4F). We compared the DEGs (increasing and decreasing) in response to SEB between each genotype pairing and identified that DR3.WT mice had the largest numbers of unique changes in comparison to DR3.IFNγ° and DR3.IL17° mice.

We also compared gene expression between genotypes treated with SEB to identify unique instances of differential gene expression (Figure 4E). Comparison of DR3.WT and DR3.IFN-γ° mice treated with SEB enriched 1,137 genes (statistically significant increasing expression) in DR3.WT and 1,768 genes in DR3.IFN-γ°. Comparison of DR3.WT and DR3.IL17° mice treated with SEB resulted in 1,054 genes enriched in DR3.WT and 1,374 genes enriched in DR3.IL17°. Comparison of DR3.IFN-γ° and DR3.IL17° mice treated with SEB resulted in 694 genes enriched (statistically significant increasing differential expression) in DR3.IFN-γ° and 661 genes enriched in DR3.IL17°. These specific DEG groupings in response to SEB may provide insight into the mechanisms behind the ablated and enhanced mortality in DR3.IFN-γ° and DR3.IL17° mice following challenge with SEB, respectively. The reminder of the RNA-seq data with the corresponding figures, tables, and their interpretation are given as a Supplementary File. The major takeaway points from exhaustive RNA-seq data are summarized below.

Expression of several Th1/Th17/Th22 cytokines, chemokines, and their receptors were significantly upregulated in the gut at 6 h corelating with serum levels of these mediators in DR3.WT mice indicating a rapid local response to SAg in the gut. An increase in the expression of STAT1 and other pro-apoptotic genes (particularly TRAIL, Fas, CASP3, and CASP8) along with decreased expression of genes associated with preservation of epithelium cell integrity/tight junctions were evident in DR3.WT mice and DR3.IL-17° mice. TNFSF10 (TRAIL), a potent inducer of apoptosis, was increased significantly in SEB treated DR3.IL17° mice in comparison to DR3.WT, and was not expressed in SEB treated DR3.IFN-γ°. Genes involved in maintenance of extracellular matrix (ECM pathways) were downregulated in DR3.WT and DR3.IL17° mice challenged with SEB. On the other hand, PBS-challenged DR3.IFN-γ° mice not only had higher baseline expression of ECM pathways compared to PBS-challenged DR3.WT and DR3.IL17° counterparts; these ECM pathways were conserved in SEB-challenged DR3.IFN-γ° mice. RNA-Seq analyses at 24 h post-SEB treatment in DR3.WT suggested waves of gene expression in comparison to 6-h treatment and changes in metabolic pathways concurrent with tissue destruction.

**Figure 5:**
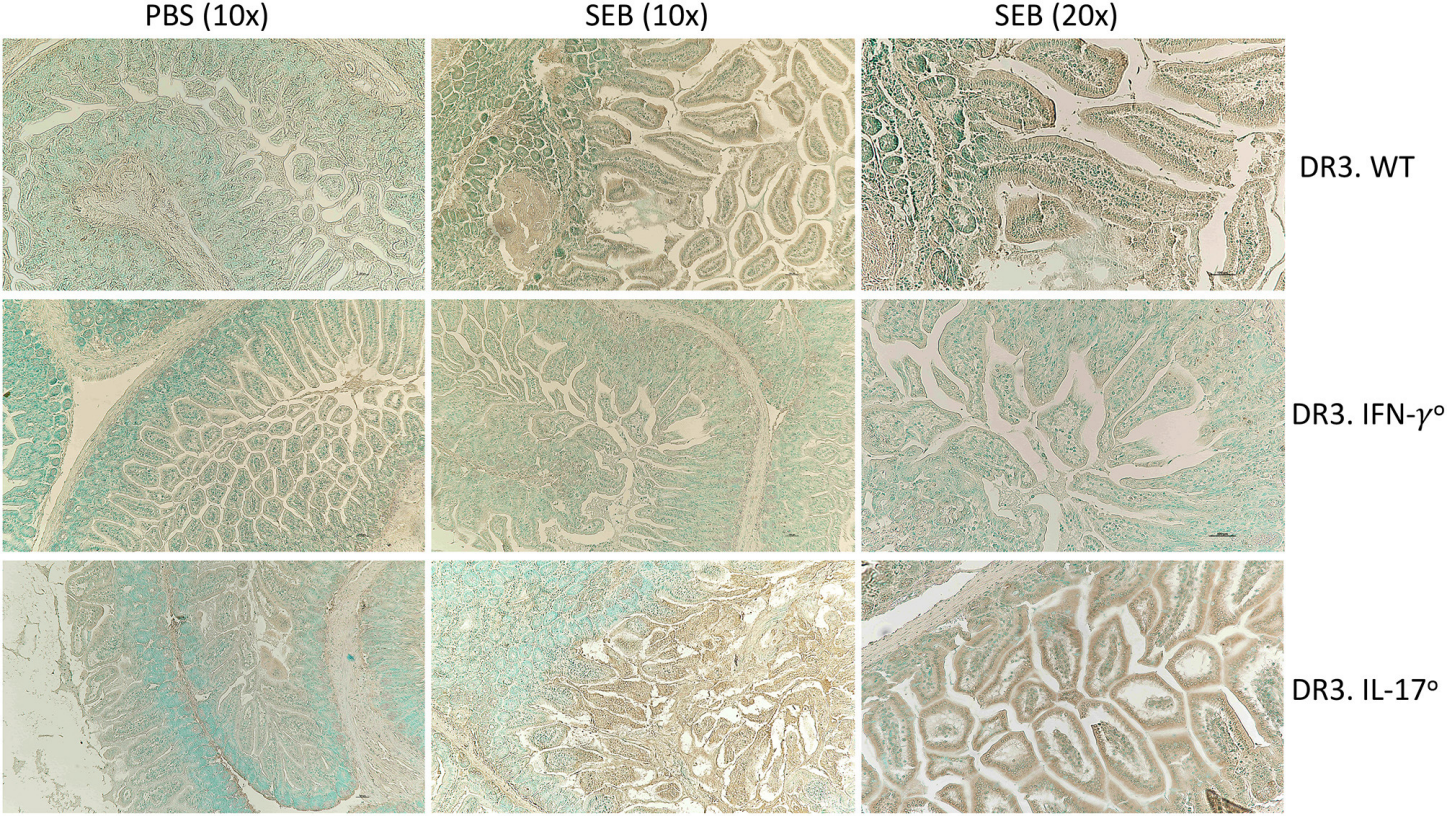
IFN-γ and IL-17A deficiency differentially modulate apoptosis in small intestines in SAg-induced CRS. HLA-DR3.WT, DR3.IFN-γ°, and DR3.IL-17° mice were challenged with 50 μg of SEB in 200 μl of PBS by intraperitoneal route. Groups of mice were euthanized at 24 h, small intestinal tissues were embedded in paraffin, processed, and thin sections were made. Extent of apoptosis in intestinal tissues collected at 24 h post SEB challenge was determined by TUNEL assay as described in methods. Representative images are shown.

Overall, (i) Histologically evident intestinal pathology in DR3.WT during T-CRS was preceded by higher expression of pro-apoptotic genes, lower expression of pro-survival genes as well as genes involved in maintenance of ECM integrity (ii) These changes were minimal in IFN-γ deficient mice that correlated with preserved small bowel architecture in DR3.IFN-γ° mice (iii) The extent of induction of pro-apoptotic genes and suppression of pro-survival genes as well as ECM pathways were much higher in DR3.IL17° mice compared to DR3.WT mice, which were supported by greater TUNEL staining (Figure 5) and higher intestinal pathology scores in these mice.

#### Real-Time Quantitative RT-PCR Confirms an Inflammatory Gene Signature

**Figure 6:**
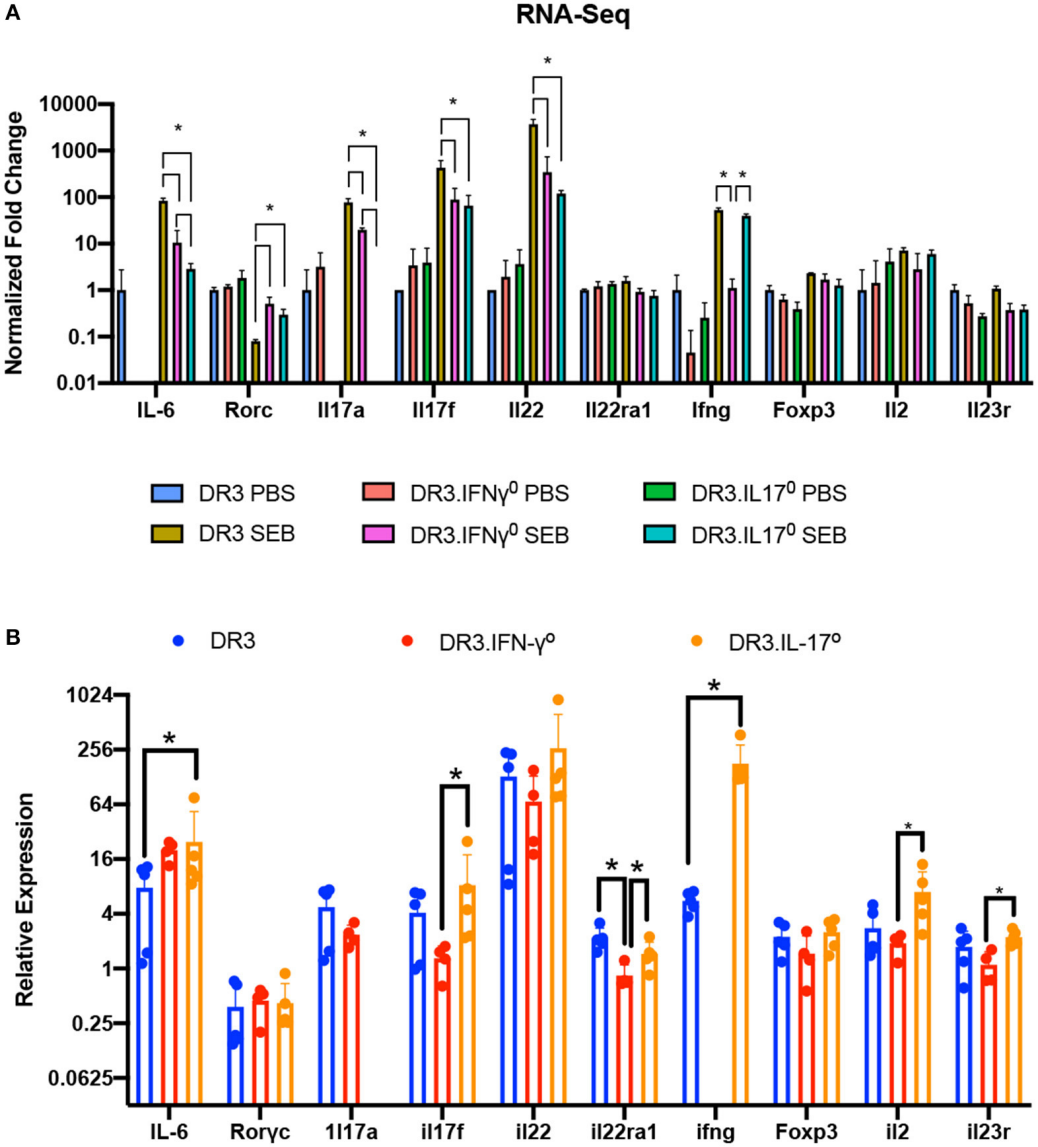
Validation of RNA-Seq gene expression changes via RT-qPCR. **(A)** Fold changes in the expression of a subset of genes involved in immune responses as determined by RNA-seq. **(B)** The expression levels of genes shown in panel A were further confirmed by RT-qPCR using the ΔΔCT method. Each bar represents mean ± SE from 4 to 5 mice. *p < 0.05.

qRT-PCR confirmed the higher expression of specific cytokine genes. Notably, the expression of IFN-γ was the highest in these mice. Most importantly, the presence of mRNA for these cytokines in the small intestines strongly suggested that cells producing these cytokines were present in the small intestines (Figure 6).

### Ruxolitinib Inhibits SAg-Induced T Cell Activation, Cytokine Production, and Proliferation *in vitro*

Complete protection of DR3.IFN-γ° mice from SAg-induced CRS and stronger upregulation of IFN-γ and STAT-1 in DR3.IL-17° mice that were highly susceptible to SAg-induced CRS suggested that blocking IFN-γ signaling could be beneficial in CRS. IFN-γ binds to the IFN-γ receptors and signals through the canonical JAK-1/2-STAT1 pathway (27, 28). While non-canonical signaling pathways have also been described (29, 30), the JAK/STAT pathway is believed to be primary signaling pathway for IFN-γ (27, 28, 30). Therefore, we hypothesized that blocking JAK/STAT pathway with JAK inhibitor could be useful in mitigating SAg-induced CRS as in certain human inflammatory diseases, malignancies, or experimental *candida* sepsis (27, 28, 30–33).

**Figure 7:**
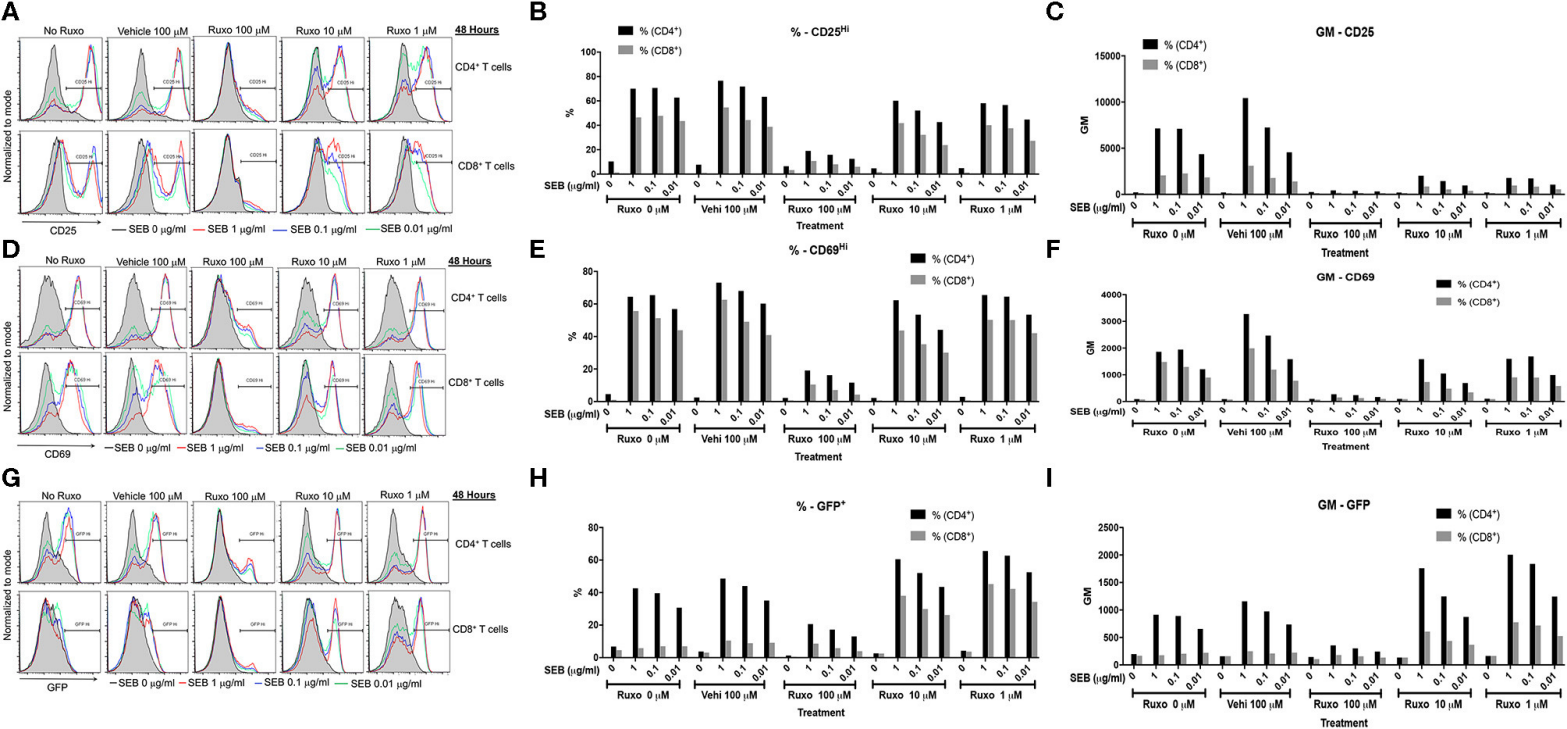
Ruxolitinib inhibits SAg-induced T cell activation *in vitro*. Splenocytes from HLA-DR3.Nur-77 GFP transgenic mice were cultured with medium alone or with indicated concentrations of SEB in the presence of ruxolitinib or vehicle for 48 h. After the incubation period, cells were washed, stained with indicated antibodies for flow cytometry. Expression of activation markers on CD4^+^ and CD8^+^-gated T cells was determined. Histogram plots, % of cells expressing the given marker and geometric mean of expression of indicated markers. CD25 **(A–C)**, CD69 **(D–F)**, and GFP **(G–I)**. Representative histograms and corresponding bar charts from one set of experiments.

Ruxolitinib has been shown to inhibit activation of human T cells *in vitro* following anti-CD3 or anti-CD3/anti-CD28 stimulation either directly (34–36) or indirectly through inhibition of dendritic cell functions (34). However, its effect on SAg-mediated T cell activation has not been tested. Therefore, we carried out a series of *in vitro* studies. First, ruxolitinib significantly inhibited SAg-induced upregulation of activation markers CD25 and CD69 on CD4^+^ and CD8^+^ T cells in a dose-dependent manner (Figure 7). T cells from DR3.GFP transgenic mice rapidly express GFP following TCR-mediated T cell activation (37, 38). We have shown previously that even SAgs are capable of upregulating GFP in T cells from DR3.GFP transgenic mice *in vivo* and *in vitro* (19) and ruxolitinib inhibited SAg-driven upregulation of GFP in T cells from DR3.GFP transgenic mice (Figure 7).

**Figure 8:**
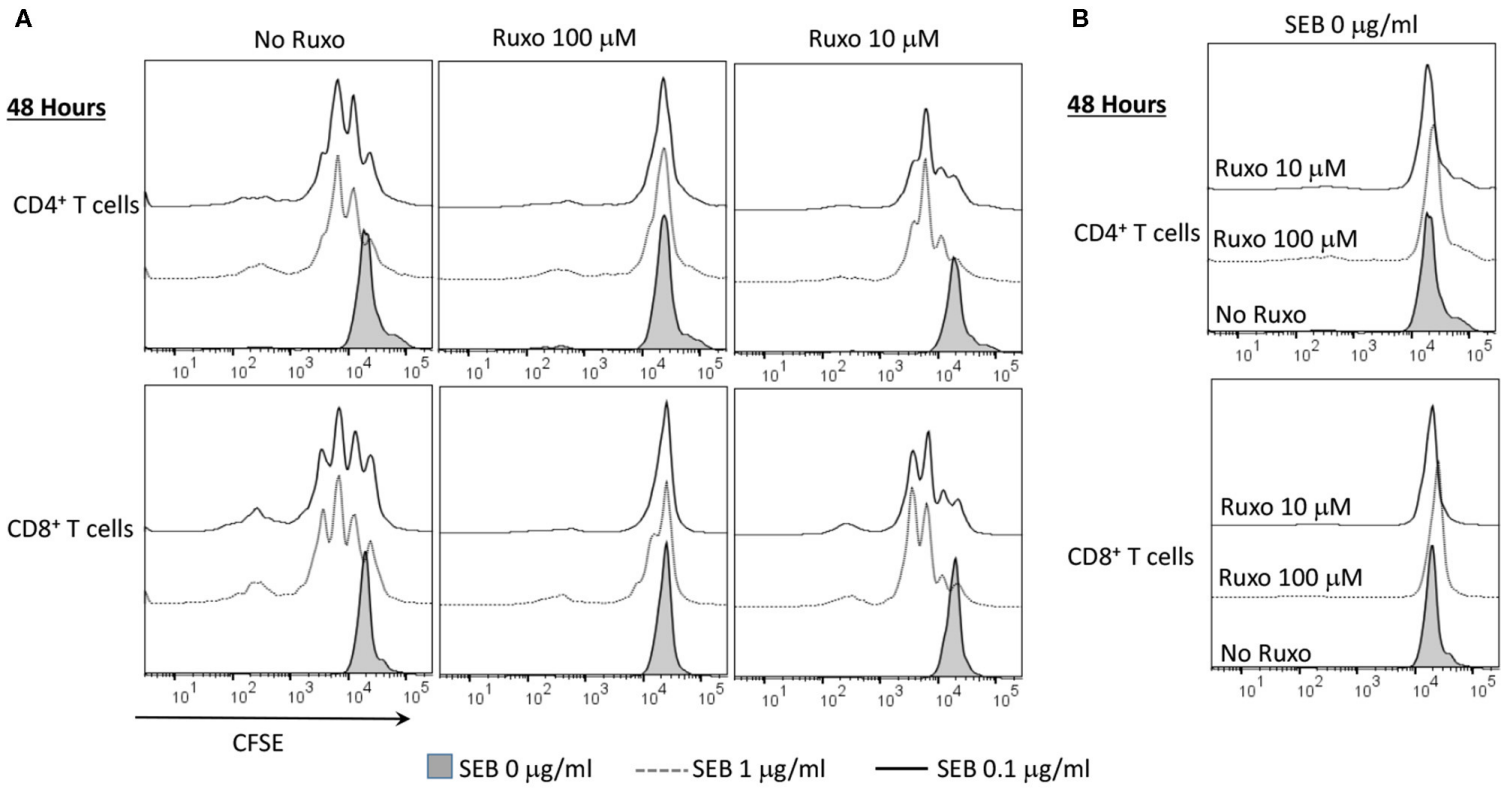
Ruxolitinib inhibits superantigen-induced T cell proliferation *in vitro*. Splenic T lymphocytes were isolated from HLA-DR3 transgenic mice using a Miltenyi negative selection kit and labeled with CFSE as described in methods. The non-T cell fraction was eluted and used as antigen presenting cells (APCs). CFSE-labeled T cells were cultured with APCs in the presence of indicated concentrations SEB **(A)** or with medium alone **(B)**, in the presence of ruxolitinib or vehicle for 48 h. After the incubation period, the cells were washed, stained with indicated antibodies and the extent of T cell proliferation was determined by flow cytometry. Similar results were obtained from 3 independent experiments.

Ruxolitinib also inhibited SAg-induced T cell proliferation in a concentration-dependent manner as determined flow cytometry-based by CFSE dilution assay (Figure 8A). Another important inference from this experiment was that ruxolitinib was not toxic at the concentrations tested because the percentage of CFSE^+^ cells were quite comparable irrespective of whether the cells were cultured only with medium, vehicle or with ruxolitinib in the absence of SEB (Figure 8B).

**Figure 9:**
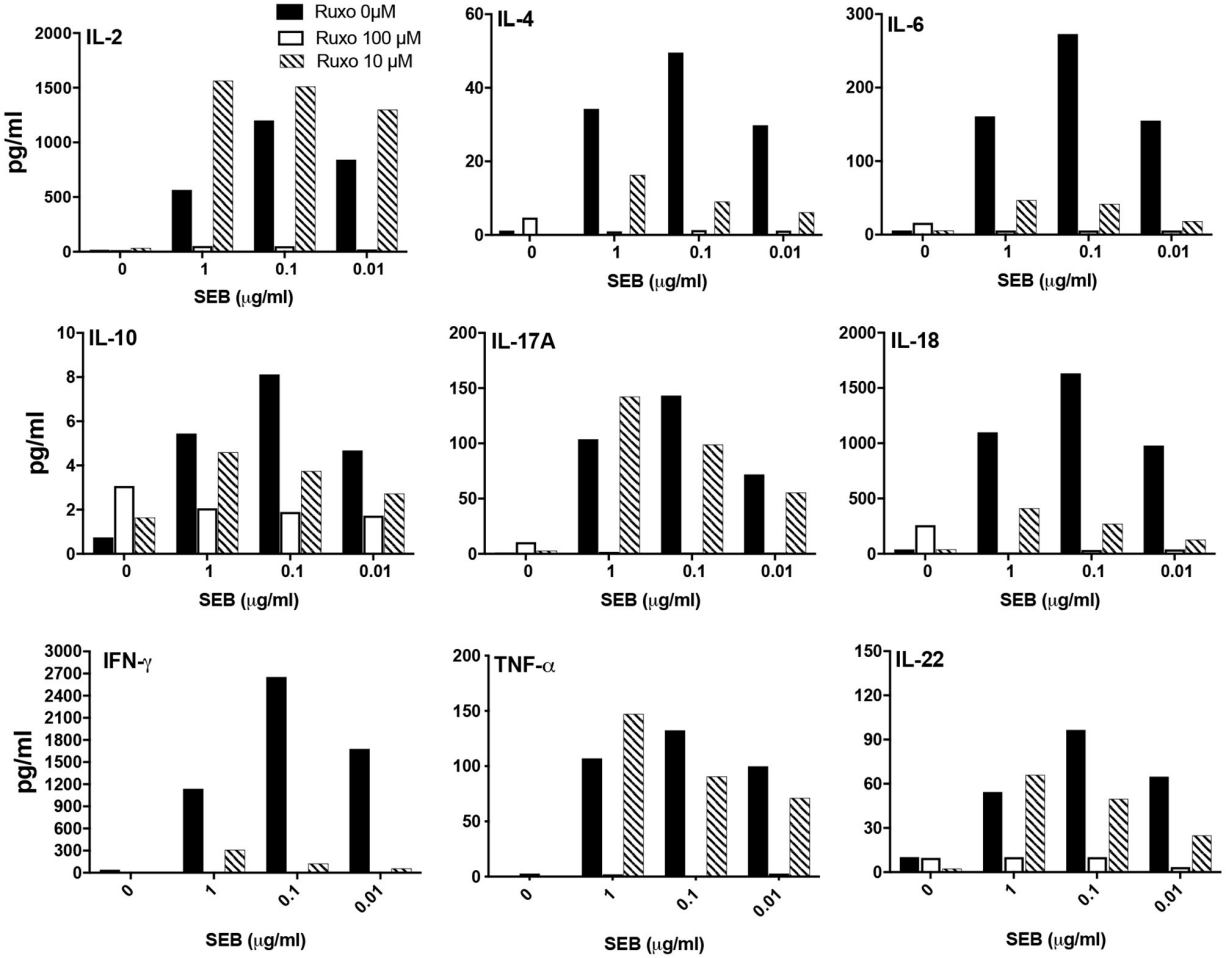
Ruxolitinib inhibits SAg-induced cytokine production *in vitro*. Splenocytes from HLA-DR3 transgenic mice were cultured with medium alone or with indicated concentrations of SEB in the presence of ruxolitinib or vehicle for 48 h. After the incubation period, the supernatants were harvested and the concentrations of indicated cytokines were determined using a multiplex assay as described in methods. Similar results were obtained from 3 independent experiments. Representative bar graphs from one experiment are shown.

We next investigated whether ruxolitinib could inhibit SAg-induced cytokine production *in vitro*. At 100 μM concentration, ruxolitinib significantly reduced the production of all the cytokines tested (Figure 9). Interestingly, at 10 μM concentration of ruxolitinib, the inhibitory effect was absent for some cytokines (e.g., IL-2), very modest inhibition on some (e.g., IL-10, IL-17A) and stronger for others (e.g., IL-4, IL-18). Remarkably, ruxolitinib completely abolished the production of IFN-γ and IL-6, the two most important cytokines implicated in the immunopathogenesis of TSS, at all the concentrations tested. Overall, based on these results, we can conclude that ruxolitinib is a potent inhibitor of SAg-induced T cell activation and cytokine production *in vitro*, particularly Th1-type cytokines, hinting a possible protective role for ruxolitinib in CRS *in vivo*.

### Ruxolitinib Attenuates SAg-Induced T Cell Activation and Cytokine Production *in vivo*

**Figure 10:**
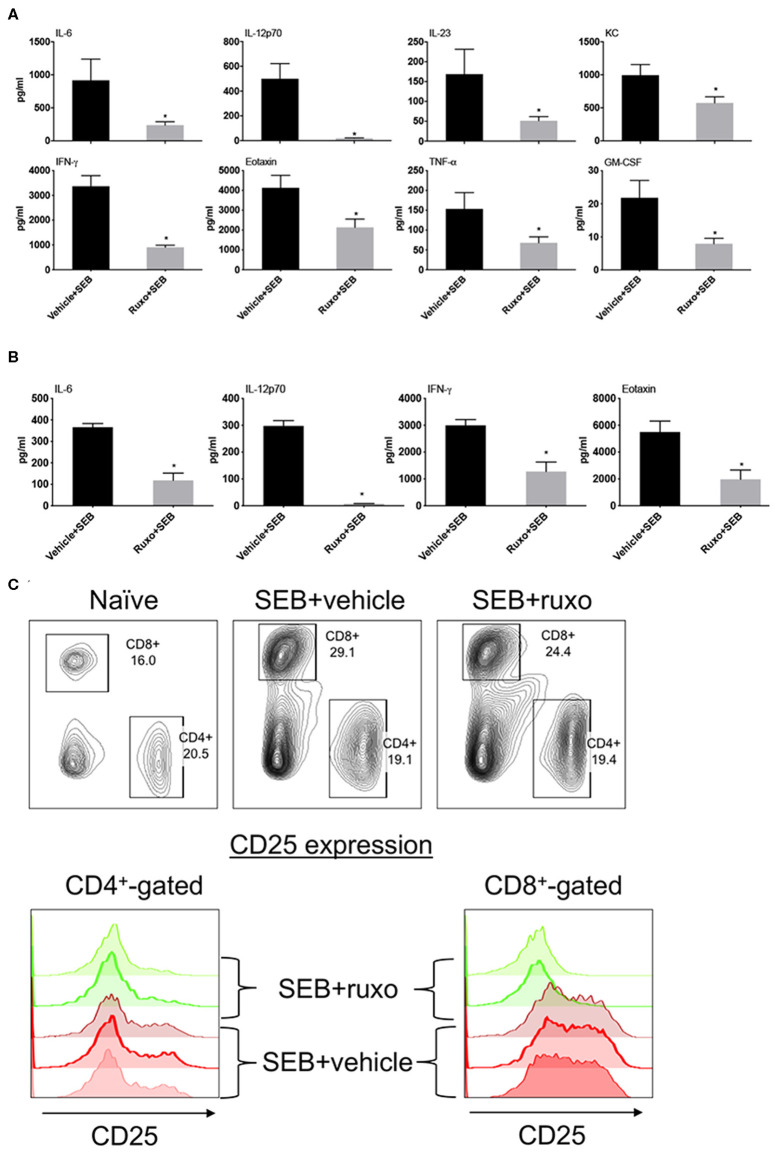
Ruxolitinib inhibits SAg-induced cytokine production and T activation *in vivo*. Age-matched HLA-DR3 transgenic mice were challenged with SEB (50 μg in 200 μl of PBS by intraperitoneal route). Mice were either pretreated with ruxolitinib or vehicle prior to SEB challenge as described in methods **(A)** or treated with ruxolitinib or vehicle immediately after intraperitoneal SEB challenge **(B)**. Animals were sacrificed 4 h after SEB challenge and the concentrations of cytokines/chemokines in individual serum samples were determined using multiplex assay kits. Only cytokines/chemokines that are significantly different between the two groups are shown for clarity. Bars represent mean ± SE from 4 to 6 mice. *p < 0.05 by unpaired *t*-test. **(C)** Mice were gavaged twice daily with ruxolitinib or vehicle from a day prior to SEB until the time of sacrifice. The animals were euthanized 48 h later. Spleens were harvested cells, mononuclear cells were isolated as per standard procedure, washed, and stained with indicated antibodies. The expression of CD25 on CD4^+^ and CD8^+^-gated splenocytes was determined by flow cytometry. Representative dot plots and histograms are shown.

Pretreatment of mice with ruxolitinib had variable inhibitory effect on cytokines. While serum levels of several cytokines including IL-2 and IL-17A were not affected similar to the *in vitro* findings, IL-6, IL-12, IFN-γ, and certain IFN-γ-dependent chemokines were significantly inhibited by prophylactic treatment with ruxolitinib (Figure 10A). When ruxolitinib was given along with SEB, fewer cytokines were inhibited. However, serum levels of IL-6, IL-12, and IFN-γ were still significantly lower suggesting that ruxolitinib could be effective even when given immediately following exposure to SAgs (Figure 10B). In addition to cytokine production, T cell activation was also inhibited by ruxolitinib treatment based on CD25 expression confirming the *in vitro* findings (Figure 10C) and supporting its potential use in SAg-induced CRS.

### Ruxolitinib Protects From Superantigen-Induced Morbidity and Mortality

**Figure 11:**
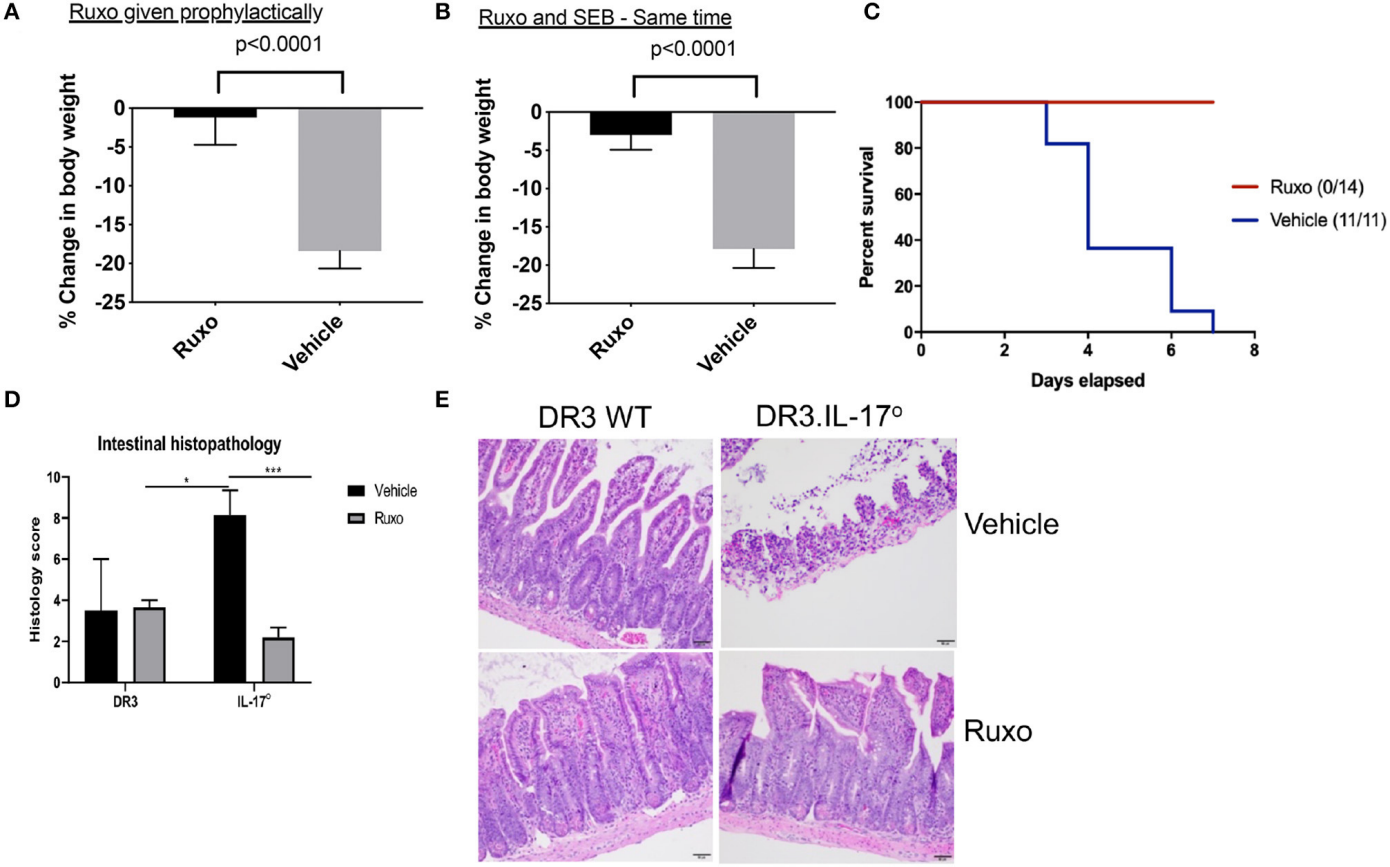
Ruxolitinib protects from superantigen-induced morbidity and mortality. HLA-DR3 transgenic mice were challenged intraperitoneally with 50 μg of SEB (reference time point 0 h). Mice were gavaged with ruxolitinib or vehicle twice daily from 1 day prior to SEB challenge **(A)** or starting immediately after SEB challenge **(B)**. Mice were closely monitored, and body weights were determined. Bars represent mean ± SE from 6 to 8 mice from each group. **(C)** HLA-DR3.IL-17 KO mice were challenged intraperitoneally with 50 μg of SEB (reference time point 0 h). Mice were gavaged with ruxolitinib or vehicle twice daily from 1 day prior to SEB challenge. Mice were closely monitored, moribund animals, and animals that lost more than 20% of their body weight were removed from the study and euthanized as per guidelines. **(D,E)** Small intestines from mice challenged with SEB and treated prophylactically with ruxolitinib were collected at necropsy (48 h), fixed in formalin, and processed routinely for H&E staining. All slides were graded by a board-certified veterinary pathologist. Sections of intestine were scored individually for amount of inflammation, loss of mucosal architecture, and epithelial damage. Scores were then summed to get a composite histologic score for that organ and animal. **(E)** shows representative images of H&E stained slides of certain fields taken at 20x magnification. DR3 Vehicle, *n* = 2; DR3 Ruxo, *n* = 3; IL-17° Vehicle, *n* = 7; IL-17° Ruxo, *n* = 10. *p < 0.05, ***p < 0.0005.

DR3.WT mice challenged with SEB and treated with the vehicle developed classical signs of CRS and continued to lose weight. Some animals lost more weight than that recommended by IACUC guidelines hence, warranting removal from study (Figure 11). However, animals challenged with SEB and treated with ruxolitinib were active, remained healthy with minimal weight loss, whether ruxolitinib was given prophylactically or immediately following systemic SEB challenge (Figures 11A,B). Ruxolitinib also completely prevented SEB-induced mortality in DR3.IL-17° mice (Figure 11C). In concordance with the survival data, HLA-DR3 and HLA-DR3.IL-17° mice challenged with SEB and treated with ruxolitinib had preserved gut architecture and had minimal signs of tissue pathology (Figures 11D,E). Taken together, our studies suggest that ruxolitinib, a potent JAK-1/2 inhibitor can be used to treat CRS caused by SAgs.

## Discussion

The morbidity and mortality associated with CRS following administration of CART therapy, T cell agonistic antibodies, immune check point inhibitors, COVID-19, or other similar conditions is attributed to failure of multiple vital organs mediated by activated T cells and the ensuing cytokine storm (1, 2, 7, 8). While it is known that cytokines contribute to MOD, it is not established whether a given cytokine plays a more lethal role compared to others. Also not understood is whether the failure of any one organ/system contributes more significantly toward lethality in CRS even though multiple organs are affected. Our murine SAg-induced CRS model provided some novel insights to these clinically relevant questions.

Rapid surge in several cytokines and multiple organ inflammation analogous to human CRS/MOD could be readily elicited in HLA-DR3 mice with SEB (1, 39, 40). Patients with CRS resulting from CART therapy have elevated levels of IL-2, IL-5, IL-6, IL-10, and particularly, IFN-γ, early on during CRS, which then returned to baseline by 2–3 days (1–3). Even in our study, there was a profound elevation in systemic levels of these T cell-derived cytokines, which fell to baseline levels by 72 h indicating similarities between all T cell-driven CRS. An early spike followed by a quick reduction in systemic levels of many cytokines even though the animals succumb to the disease days later suggested complex nature of the disease and that, the therapeutic interventions for CRS should start as early as possible before the cytopathogenic molecular pathways become irreversible.

The major hallmark of severe CRS with CART therapy or following administration of T cell-stimulatory antibodies is MOD (1, 2). Even though there is no underlying infection, an uncontrolled or overactivation of T cells in these conditions leads to MOD (1, 2, 41–43). Similar findings in HLA-DR3 mice challenged with SEB support the notion that cytokines or other inflammatory mediators produced by activated T cells are the major contributors to MOD, whether they are activated by agonistic antibodies, tumor antigens, or SAgs. Our study also suggested that even though multiple organs fail in SAg-induced CRS, immunopathology to the small intestines might be the most important cause for death. This is an important finding because small bowel pathology is not a common feature of experimental SAg-induced CRS in standard laboratory mice expressing mouse MHC class II molecules. CRS is milder in standard laboratory mice because SAgs bind weakly to mouse MHC class II molecules. Moreover, the use of hepatotoxic sensitization agents such as D-galactosamine in standard laboratory mouse models of SAg-induced CRS, directs the inflammatory insult to the liver, resulting in rapid mortality primarily due to hepatic dysfunction (15, 44, 45). Since such sensitizing agents were not used in our model, it enabled the appreciation of inflammation and immunopathology in other organs, particularly to the small intestines.

The significance of gut failure in fatal MOD in SAg-induced CRS is in line with clinical and other experimental evidences that the failure of normal intestinal functions plays a significant role in the immunopathogenesis of MOD (46–49). It is even believed that “gut failure is a motor that both drives and perpetuates multiple organ dysfunction” (50) and numerous clinical studies have underscored the significance of measuring gastrointestinal functions as predictors of mortality in several serious systemic inflammatory diseases such as sepsis (51–53).

Mechanistically, the gut is a major secondary lymphoid organ rich in CD4^+^ and CD8^+^ T cells. Therefore, we believe that these gut-resident T cells are rapidly activated by SAgs in this study (or agonistic antibodies or checkpoint inhibitors during cancer immunotherapy), produce copious amounts of cytokines, thereby causing profound immunopathology to the small intestines. The abundance of mRNA for several cytokines, chemokines, and inflammatory mediators in the intestines in the SAg-induced CRS model in the current study support this model (54–56). Further, studies on DR3 WT mice showed strong upregulation of pro-apoptotic molecules, lower expression of pro-survival genes along with reduction in expression of genes associated with preservation of extracellular matrix integrity during CRS. Together, these molecular changes led to microscopically quantifiable intestinal pathology. Extensive intestinal epithelial cell damage likely resulted in loss of epithelial cell functions (such as nutrient absorption, fluid/electrolyte balance) thus resulting in death due to malnutrition as well as dehydration. Further, a loss of intestinal epithelial cell integrity could have resulted in translocation of microflora from the gut into the blood stream resulting in septicemia. Additional studies are warranted to tease out the exact causes of mortality during CRS, and whether antibacterials combined with parenteral nutrition as well as adequate fluid management could lower mortality.

Our studies also identified opposing roles for IFN-γ and IL-17A in inducing small bowel pathology in CRS, with IFN-γ being pathogenic and IL-17A being protective. IFN-γ has been shown to induce extensive small bowel pathology *in vivo*, and in intestinal organoids *in vitro*, by multiple mechanisms including through induction of apoptotic pathways and internalization of epithelial tight junction proteins (57–59). IFN-γ can also regulate intestinal epithelial cell homeostasis through serine-threonine protein kinase AKT-beta-catenin and Wingless-Int (Wnt)-beta-catenin signaling pathways (58–60). However, robust upregulation of STAT1 in the intestines and significant protection conferred by the JAK-1/2 inhibitor, ruxolitinib suggested that IFN-γ -JAK-1/2- STAT1 pathway may play a major role in inducing small intestinal immunopathology in SAg-induced CRS. Even though STAT1 is not directly involved in apoptosis, it is a well-known inducer of apoptosis via several other pathways that are involved in apoptosis (Bak, Bcl-2, Bcl-XL, Casp1, Casp8, Trail, DR5, FasL, Fas), which are also upregulated in the intestines in our model (61–63). Hence, ruxolitinib could have directly blocked IFN-γ mediated, JAK-1/2 driven upregulation of these pro-apoptotic molecules in the intestinal epithelial cells thereby protecting them. However, ruxolitinib was also able to significantly inhibit SEB-induced production of pro-inflammatory cytokines, especially Th1 cytokines such as IL-6, IL-12, and IFN-γ itself, both *in vitro* and *in vivo* (either prophylactic or immediately after induction of CRS). Hence, the protective effects of ruxolitinib could also be indirectly through reducing the production of pathogenic cytokines by the immune system. Further studies are ongoing to identify the primary targets of ruxolitinib, whether the intestinal epithelial cells or the immune cells.

Unlike IFN-γ, IL-17A played a protective role in acute SAg-induced small bowel pathology in our model contrary to a recent publication (64). This is also a novel finding because a majority of the studies have only addressed the role of IL-17A in various models of chronic intestinal inflammation, particularly of the large intestines, not involving SAgs. Even in these studies, IL-17A has been shown to play contradictory roles. Some studies have shown that IL-17A-deficient mice have exaggerated intestinal inflammation, whereas others have shown that IL-17A-deficiency in mice is associated with less severe intestinal inflammation and immunopathology [(65–70) and reviewed in (71)]. However, in our SAg-induced CRS model, IL-17A clearly played a protective role.

A gut-protective role for IL-17A has been described in an anti-CD3-induced, T cell-mediated acute intestinal inflammation in mice (67). Unexpected exacerbation of Crohn's disease following treatment with human anti-IL-17A monoclonal antibody in patients with inflammatory bowel disease and *de novo* onset of inflammatory bowel diseases in psoriatic patients following anti-IL-17 antibody therapy support a gut-protective role of IL-17 even in humans (72, 73). However, the mechanisms by which IL-17A plays a gut protective role has not been elucidated in detail and many pathways have been proposed (74). However, as discussed earlier, a vast majority of these studies have only investigated the role of IL-17A in the context of colitis and not acute in small bowel inflammation. One study proposed that IL-17A plays suppressive roles in spontaneous colitis in conjunction with IL-10 through myeloid derived suppressor cell (MDSC) and inducible nitric oxide synthase (iNOS) (75). Elevated STAT1 expression combined with higher expression of other pro-apoptotic models in DR3.IL-17° mice, and significant protection conferred by ruxolitinib in DR3.IL-17° mice in our model suggested that IL-17A plays a gut-protective role by antagonizing the pathogenic functions of IFN-γ by inhibiting its signaling, reducing apoptosis, and thereby preserving intestinal epithelial cell functions. Further studies are needed to make definitive conclusions. Overall, hyperactivation of IFN-γ-dependent inflammatory pathways in the absence of IL-17A support the emerging concept that IL-17A might play a protective role at least in the mucosal immunity [reviewed in (76)].

Ruxolitinib is an orally bioavailable Janus kinase (JAK) inhibitor with potential antineoplastic and immunomodulating activities. It was approved for clinical use to treat primary myelofibrosis and later for other malignancies. Subsequently, ruxolitinib, and other JAK inhibitors were approved for clinical use in a variety of inflammatory conditions including alopecia areata (33). JAK1/2 inhibitors are expected to play a beneficial role even in COVID-19 and clinical trials are ongoing to evaluate the benefits of JAK1/2 inhibitors in CRS associated with COVID-19 (77). Since IL-17A signaling does not involve JAK-1/2 pathways, preserving the gut-protective IL-17A signaling pathways, and inhibiting only the pathogenic IFN-γ-driven JAK-1/2 pathways using JAK-1/2 inhibitors is likely a beneficial approach for treating CRS. Further studies are required to delineate the molecular pathways by which ruxolitinib protects from lethal CRS, narrow down the therapeutic window of ruxolitinib, therapeutic time window, and the duration of treatment for CRS.

The other important observation made in the study is the high expression of IL-22 in the intestinal tissue during SAg-induced CRS. IL-22 is a newly identified cytokine belonging to the IL-10 family (78–80). IL-22 is primarily produced by activated CD4^+^ T cells. However, the IL-22 receptor is expressed exclusively on the epithelial cells. Similar to IL-17A, the role of IL-22 in intestinal inflammation is controversial; some studies indicating a protective role, while others pointing to a pathological role (78–81). Our SAg-induced CRS in HLA-DR3 transgenic mice could be useful for investigating the role of IL-22 in the immunopathogenesis of MOD.

Overall, our study suggested that IFN-γ-dependent small bowel pathology played a significant role in the immunopathogenesis of SAg-mediated T cell-driven CRS and that IL-17A played a protective role. In addition to CRS, our findings could be applicable to serious infections such as pneumonia and sepsis caused by *S. aureus* and *S. pyogenes*. As many virulent *S. aureus* and *S. pyogenes* strains associated with serious infections produce potent or novel SAgs (82, 83), SAg-induced IFN-γ might contribute to the immunopathogenesis of MOD at least in a subset of patients with these bacterial infections and blocking IFN-γ signaling with JAK inhibitors might be useful (84–86).

## Data Availability Statement

The datasets generated for this study can be found at the Gene Expression Omnibus (GEO) under accession GSE130125.

## Ethics Statement

The animal study was reviewed and approved by the Virginia Tech Institutional Animal Care and Use Committee and the Office of Laboratory Animal Welfare. The assurance number is A-3208-01.

## Author Contributions

GR: conception or design of the work. SDK, BM, MS, BK, RM, AR, SVK, SC-O, YS, JF, JK, and GR: acquisition, analysis, or interpretation of data. SDK, SC-O, JK, and GR: drafting and revising. All authors contributed to the article and approved the submitted version.

## Conflict of Interest

The authors declare that the research was conducted in the absence of any commercial or financial relationships that could be construed as a potential conflict of interest.
