# Supplementary material for: “Small” Intestinal Immunopathology Plays a “Big” Role in Lethal Cytokine Release Syndrome, and Its Modulation by Interferon-γ, IL-17A, and a Janus Kinase Inhibitor
Source: Front Immunol. 2020 Jun 26;11:1311. doi: 10.3389/fimmu.2020.01311 (PMC7333770; doi:10.3389/fimmu.2020.01311)
Supplement: Supplementary file 2 [file Data_Sheet_1.pdf]

RNA-Seq supplemental Data:

1. Enrichment of annotated immune signaling pathways in DR3.WT mice early on during SAg-induced CRS.

We first looked for pathways enriched by DEGs associated with SAg-induced CRS in DR3.WT mice in comparison to PBS treatment. Enrichment analysis was done by overlaying DEGs onto the Reactome protein-protein interaction networks (Supplementary Figure 4A). Reactome pathways allow for enrichment analysis of several expertly curated repositories include KEGG, Reactome, PantherDB, *etc*. Of the 2,311 identified DEGs, 859 were found in the Reactome protein-protein interaction network (Supplementary Figure 4A, Supplementary Table 3). These 859 nodes were connected by 4,020 edges (4.68 edge/node ratio), indicating increasing expression of densely linked pathways. Pathway analysis indicated 58 pathways enriched by DEGs increasing in expression post-treatment ($p < 0.01$, FDR, Supplementary File1 1 and Supplementary Table 3). DEGs increasing in expression enriched a large number of immune signaling pathways specifically of interest included: Cytokine-cytokine receptor interaction (Kegg), Chemokine signaling pathway (K), signaling by interleukins (Reactome), TNF-α (K), JAK-STAT (K), IFNα/β (R), IL-23 (NCI-database), TNFR2 non-canonical NFκB (R), and NFκB signaling pathways (K) (Supplementary Figure 4B, Supplementary File 1). Based on pathway enrichment of cytokine-cytokine receptor interaction, we identified 20 cytokines (5 of which are members of the TNF superfamily) and 22 chemokines to be differentially expressed, some by over 10-fold (Supplementary Figure 4CD). Both IFN-γ and IL-17A were DEGs in response to SEB, further suggesting their primary importance in immune signaling. Of additional significant note is the fact that IL-22 is the DEG with greatest fold change in response to SEB (from 0 counts in the PBS control to 3,167 in response to SEB).

Manual evaluation of these differentially expressed chemokines indicates a preference for neutrophil, eosinophil, activated T-cells, and monocyte recruitment. Evaluation of differentially expressed cytokines suggests a general pro-inflammatory response with specific expression of cytokines driving Th1/Th17/Th22 responses in response to SEB. Further gene expression analysis of known transcription factor regulators driving these responses reiterated the pronounced Th17/Th22 response via STAT3 activation (Akdis et al., 2012) and Th1-type response via STAT1 activation (Horvath, 2004) (Supplementary Figure 4E). Though immune signaling pathways made up a large portion of the enriched pathways, a large number of DEGs with increasing expression also enriched Apoptosis (Supplementary Figure 3C), rRNA processing, ribosome biogenesis, spliceosome function, HIF-1 signaling, dissolution of fibrin clots, Mitotic G1-G1/S phases, and tRNA aminoacylation (Supplementary File 1). These finds suggest key shifts in fundamental biological processes as well as enhanced protein production.

We then analyzed DEGs decreasing in expression response to SEB to identify enriched pathways and processes that may be halted or down regulated. Of the 3,118 DEGs decreasing in expression 754 were identified in the Reactome protein-protein interaction network. These 754 nodes were connected by 1,907 edges (2.57 edge/node ratio). Enriched pathways associated with decreasing gene expression of particular note were Gastrin-CREB signaling pathway via PKC and MAPK(R), Rap1 signaling, bile secretion, lipid digestion, mobilization, and transport, fluid secretion, and cAMP signaling, PI3K-Akt signaling, and ECM matrix organization (Supplementary Figure 3F and Supplementary File 1). Our RNA-Seq results suggest enrichment

of pathways related to the breakdown of the epithelium, halting of major functions of the small intestine (bile and fluid secretion, and gastric hydrochloric acid production), and lipid breakdown in response to SEB. Enrichment of pathways related to the breakdown of the epithelium, halting of major functions of the small intestine (bile and fluid secretion, and gastric hydrochloric acid production), and lipid breakdown indicated impending gut pathology that could be histologically confirmed at 24 and 48 hours (Figure 2).

2. DR3.IFN-γ^o and DR3.IL17^o mice have a generally diminished expression of cytokines/chemokines during SAg-induced CRS.

Our initial analysis suggested an increase in expression of genes encoding cytokines and chemokines in response to SEB for DR3.WT mice. We then looked at differential expression of cytokines and chemokines in DR3.IFN-γ^o and DR3.IL17^o mice in response to SEB as well as in comparison to DR3.WT mice as this may provide insight into the decreased/increased mortality (Supplementary Figure 5). We looked at both unique occurrences of differential expression in response to SEB as well as differential expression between genotypes post SEB treatment (Supplementary Figure 5). Only 10 of the 22 cytokines and 10 of the 22 chemokines that were identified to be differentially expressed in response to SEB in DR3.WT were also differentially expressed for DR3.IFN-γ^o mice (Supplementary Figure 5AB). This suggested a significantly ablated response in immune signaling in DR3.IFN-γ^o mice. Direct comparison of DR3.IFN-γ^o and DR3.WT mice indicated 12 cytokines and 10 chemokines that were enhanced in expression for DR3.WT mice. Expression of only 2 cytokines, IL-13 and Tnfsf9 (also called as 41BB ligand), and 1 chemokine, CXCL13, was increased in DR3.IFN-γ^o mice (Supplementary Figure 5C). Overall our analysis suggested decreased expression of cytokines and chemokines in the absence of IFN-γ rather than an induction of several novel cytokines and chemokines.

Analysis of cytokine/chemokine expression in response to SEB for *DR3.IL17^o* mice also suggested an ablated response in comparison to DR3.WT mice. 8 cytokines and 13 chemokines were expressed at a lower level in DR3.IL17^o mice treated with SEB in comparison DR3.WT mice. No chemokines were uniquely differentially induced by SEB in DR3.IL-17° mice, while three cytokines (IL-1β, IL-7, and TNFSF10) were unique in their differential expression. Interestingly, IL1β and IL-7, were not considered differentially expressed between DR3.WT and DR3.IL17^o mice treated with SEB, while TNFSF10 (TRAIL) was increased by more than 1.5-folds in DR3.IL17^o mice (Supplementary Figure 5A).

Finally, we looked at the instances of cytokine/chemokine differential expression between DR3.IFN-γ^o and DR3.IL17^o mice in response to SEB (Supplementary Figure 5C). Only 3 cytokines and 5 chemokines had enhanced expression in DR3.IFN-γ^o mice treated with SEB, while 4 cytokines and 6 chemokines had enhanced expression in DR3.IL17^o mice treated with SEB. A majority of cytokines and chemokines with enhanced expression for either DR3.IFN-γ^o and DR3.IL17^o mice had similar expression levels to that of DR3.WT mice. This suggested though the loss of *IFN-γ* or *IL17* resulted in specific instances of decreased cytokine/chemokine expression. Of all the cytokines and chemokines analyzed, only TNFSF10 (TRAIL) had enhanced expression that was unique to DR3.IL17^o in comparison to DR3.WT and DR3.IFN-γ^o.

3. DR3.WT and DR3.IL17^o mice treated with SEB are enriched for apoptosis pathways in comparison to DR3.IFN-γ^o mice treated with SEB.

Our analysis of genes differentially expressed for DR3.WT mice in response to SEB resulted in the enrichment of several immune signaling pathways, of particular note the apoptosis pathway (K). We repeatedly found the apoptosis pathway to be enriched amongst differentially expressed genes from DR3.IL17^{o} mice and DR3.WT mice treated with SEB in comparison to DR3.IFN-γ^{o} mice treated with SEB (Supplementary File 1). Of particular interest was the overexpression of TRAIL, Fas, CASP3 and CASP8 in DR3.WT in comparison to DR3.IFN-γ^{o} suggesting increased apoptosis, and higher expression of perforin and granzyme B suggested cell death induction by cytotoxic lymphocytes (Supplementary Figure 6A). We found that DR3.IL-17° mice had significantly higher expression of key proteins associated with the induction of apoptosis (Trail, STAT1), and lower expression of pro-survival genes (Bcl-XL, FAP1, Gadd45, IAP) in comparison to DR3.WT mice (Supplementary Figure 7, Supplementary Figure 6). Interestingly STAT1, though not included in the apoptosis pathway, is a well-known inducer of apoptosis via several pathways (Bak, Bcl-2, Bcl-XL, Casp1, Casp8, Trail, DR5, FasL, Fas). STAT1 and several other markers (Fas, FasL, Trail) were differentially expressed to a significantly higher level in DR3.WT and DR3.IL-17° mice, whereas expressed at significantly lower levels in DR3.IFN-γ^{o} mice treated with SEB (Supplementary Figure 7). Overall, our findings suggest a lack of expression of genes associated with pro-apoptosis in the small intestines of DR3.IFN-γ^{o} mice following challenge with SEB, whereas both DR3.WT and DR3. IL17^{o} mice highly expressed several pro-apoptotic genes following challenge with SEB. In addition, DR3. IL17^{o} mice failed to adequately express inhibitors of apoptosis, resulting in net increase in apoptosis in the small intestines, which correlated with histopathological findings. TUNEL staining of small intestinal sections (24-hour time point) showed evidence of apoptosis in DR3.WT and DR3.IL-17° mice, with stronger positive signals in the latter (Figure 5). Sections from DR3.IFN-γ^{o} mice had the least TUNEL staining, correlating with H&E findings.

We then thought to cluster immunologically relevant genes (IRGs) across all 6 conditions/genotypes to gain further insight into the type of immune response as immunity is associated largely with the induction of various forms of programmed cell death (Supplementary Figure 7). DR3.WT and DR3.IL17^{o} mice treated with SEB had significantly increased in IFN-γ gene expression by over 50 and 150-fold, respectively (Supplementary Figure 5A). Interestingly, many interferon-inducible genes, including interferon-regulatory factors (IRF 1, 3, 5, 7, and 8) and Immunity-related GTPase family M protein 1 (Irgm1) were found to be strongly expressed in DR3.IL17^{o} mice further supporting the elevated levels of IFN-γ activity in these mice compared to DR3.WT mice (Supplementary Figure 7E).

Haptoglobin, an abundant plasma protein that captures hemoglobin and associated with sepsis and inflammatory bowel disease in humans, was also highly upregulated in DR3.IL17^{o} mice (156-folds compared to 52-folds in WT mice and 24-folds in DR3.IFN-γ^{o} mice) (Vanuytsel et al., 2013; Andersen et al., 2016). Z-DNA binding protein 1 (ZBP1), is another host protein recently recognized as an innate immune sensor regulating activation of both programmed cell death and inflammation (Kuriakose and Kanneganti, 2018). Interestingly, expression of ZBP1 was 5-6-times higher in the intestines of DR3.IL17^{o} mice suggesting a potential role for this molecule in acute intestinal inflammation elicited by SEB. The expression of another protein involved in innate immune signaling and IFN pathway, namely the nucleotide-binding domain (NBD) leucine-rich repeat (LRR) containing protein, NLRC5 was also higher in DR3.IL17^{o} mice (3-4 times) (Davis et al., 2011). Overall, significantly higher mortality in DR3.IL-17° mice could be associated with STAT1 and TRAIL hyper expression along with upregulation of several pro-

apoptotic and pro-inflammatory genes. Conversely, lower expression of STAT1, TRAIL, pro-apoptotic genes, combined with elevated expression of anti-apoptotic genes in DR3.IFN-γ° mice could have been important for the enhanced survival of DR3.IFN-γ° mice.

4. DR3.IFN-γ° mice have heightened extra-cellular matric (ECM) integrity in response to SEB in comparison to DR3.WT mice.

The downregulation of ECM proteins in response to SEB in DR3.WT mice, provoked us to look for similar pathways enriched in DR3.IFN-γ° and DR3.IL17° in response to SEB. Both DR3.IFN-γ° and DR3.IL17° mice had a large number of genes that were also differentially decreased in expression in response to SEB suggesting the breakdown of the extracellular matrix in response to SEB. Quite surprisingly, naïve DR3.IFN-γ° mice had a subset of differentially expressed genes that enriched the preservation and fortification of the ECM in comparison to naïve DR3.WT and DR3.IL-17° mice. These genes are associated with the formation and maintenance of elastic fibers (ELN, FBN2), fibrillar collagen (COL3A1), connective tissue microfibrils (FBN2, MFAP5), and matrix-associated proteins (ADAMTS2, SPARC) (Supplementary File 1, Supplementary Figure 8A). Analysis of enriched genes pathways for DR3.IFN-γ° mice treated with SEB resulted in enrichment of pathways associated with extracellular matrix organization in comparison to both DR3.WT and DR3.IL17° mice treated with SEB (Supplementary Figure 8B). This indicated the breakdown of ECM was greater for DR3.WT and DR3.IL17° mice. Overlay of gene expression onto the KEGG ECM-receptor pathway indicated the down regulation of a large number of genes in response to SEB across all genotypes; however, a subset of genes encoding collagen, laminin, and integrin were specifically increased in expression for DR3.IFN-γ° mice treated with SEB. Our analysis suggests DR3.IFN-γ° mice had enhanced expression for genes encoding components of the ECM prior to SEB treatment, and uniquely expressed genes that aided in the preservation of ECM integrity in response to SEB.

We then sought to study the gene expression changes in the small intestines of DR3.WT mice at 24 hours post-challenge with SEB in order to determine the responses in the major target organ over time. We performed RNA-Seq analysis on samples in an identical manner as for 6-hour time point. Analysis was done using DR3.WT PBS and 6-hour SEB-treated samples as normalization factors. Based on this analysis, we identified 4,750 (2,213 increasing, 2,537 decreasing) genes that were differentially expressed at 24 hours in comparison to control via DESeq2 (Supplementary Figure 9A) and 3,867 (2,268 increasing, 1,599 decreasing) genes differentially expressed at 24 hours in comparison to 6 hours. Overall, the gene expression fell into 4 patterns (Supplementary Figure 9BC): (i) A wave like phenomenon where gene expression initially increases/decreases by 6 hours and then decreases/increases by 24 hours. (ii) Increase/decrease consistently from 0 to 6 to 24 hours. (iii) Increase/decreases from 0 to 6 hours and then hold steady from 6 hours to 24 hours. (iv) No changes from 0 to 6 hours and then increase/decrease from 6 to 24 hrs. These gene groupings were then used for enrichment analysis in addition to DEG groupings between 0 and 24-hour and 6- hour and 24-hour post-SEB treatment.

Enrichment of Reactome Pathways via DEG groupings provided novel insights into the functions of genes with a wave of differential expression. Group A encoded a subset of cytokines and chemokines, and IL-23-mediated signaling events. Interestingly, both IL-10 and TNF-α

were both a part of this grouping suggesting elevated signaling for pro- as well as anti-inflammatory pathways. Group B encoded genes functioning in ribosome biogeneisis and rRNA processing as well as a subset of cytokine-cytokine interactions and chemokine signaling pathways. Interestingly several genes enriched genes associated with mitosis and cell cycle regulation. This suggested an immediate response to initiate protein production and halting of basal cellular functions Group C, indicated shut down of pathways associated with carbohydrate, bile, and lipid digestion and absorption as well as insulin secretion, but were then reactivated. Group E consisted of several pathways previously identified for DEGS between naïve and SEB treatment at 6 hours (TNF-α, JAK-Stat signaling, NFκB signaling, apoptosis, etc.) indicating these gene expression changes do not decrease but remained elevated. Group F, genes only differentially expressed from 6 to 24 hours, was enriched for genes and pathways involved in repairing extracellular matrix organization, NK mediated cytotoxicity, Th1/Th2/Th17 differentiation, IL-12 signaling suggesting additional cytotoxicity, an enhancement of mixed T cell differentiation as well as attempt by the tissue to limit the damage and start the tissue repair process (Supplementary Figure 9). Analysis of genes in Group G, genes with a decreased differential expression start at hour 6 to 24, indicated shut down of components of the TCA cycle and a near complete shutdown of respiratory electron transport Complex I (Supplementary Figure 9E), propanoate metabolism, and Mitotic G2-G2/M phases. Grouping H suggested a decrease in expression of genes associated with several growth factor pathways (PDGF, EGFR, NGF, and VEGF), Wnt, mTOR, PPAR signaling, and regulation of lipolysis. Overall, the additional 24-hour time point provided us added insight into the continuity of several inflammatory functions identified at 6 hours as well as a wave like gene expression pattern for genes that primed for protein biosysnthesis or prevented absorption or digestion of molecules. Of particular importance is the shutdown of the TCA cycle at succinate dehydrogenase and pyruvate dehydrogenase and mitochondrial electron transport from 6 to 24 hours indicating a shift in energy away from oxidative phosphorylation and a push into cell death and inflammation (Supplementary Figure 9E).

Overall, the findings from extensive RNA-seq analyses can be summarized as follows (i) Histologically evident intestinal pathology in DR3.WT during CRS was preceded by higher expression of pro-apoptotic genes, lower expression of pro-survival genes and suppression of genes maintaining ECM integrity (ii) These changes were minimal in IFN-γ deficient mice that correlated with preserved small bowel architecture in DR3.IFN-γ^o mice (iii) The extent of induction of pro-apoptotic genes and suppression of pro-survival genes as well as ECM pathways were much higher in DR3.IL17^o mice compared to DR3.WT mice, which were supported by greater TUNEL staining and higher intestinal pathology scores in these mice.

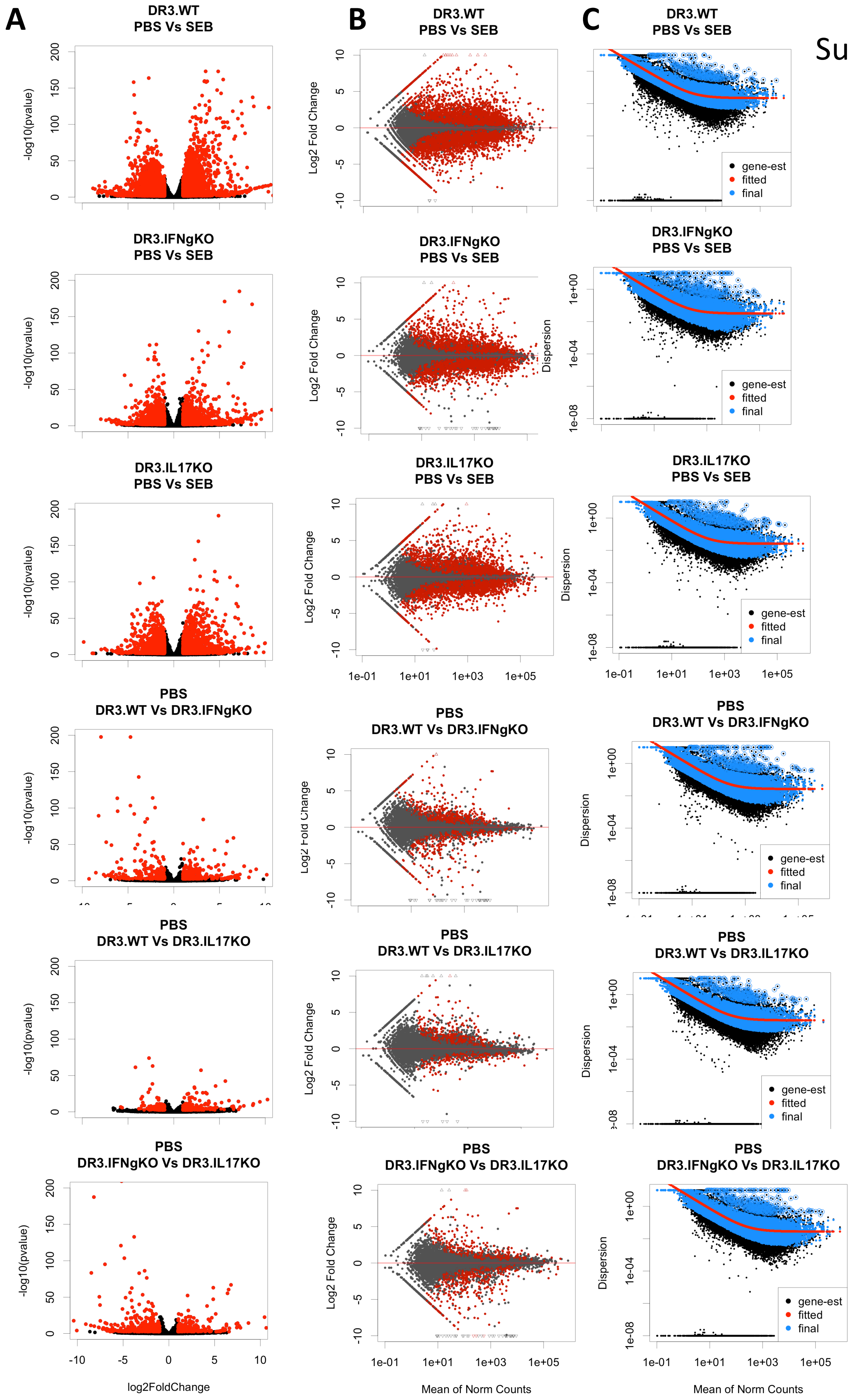

Supp. Figure 1.

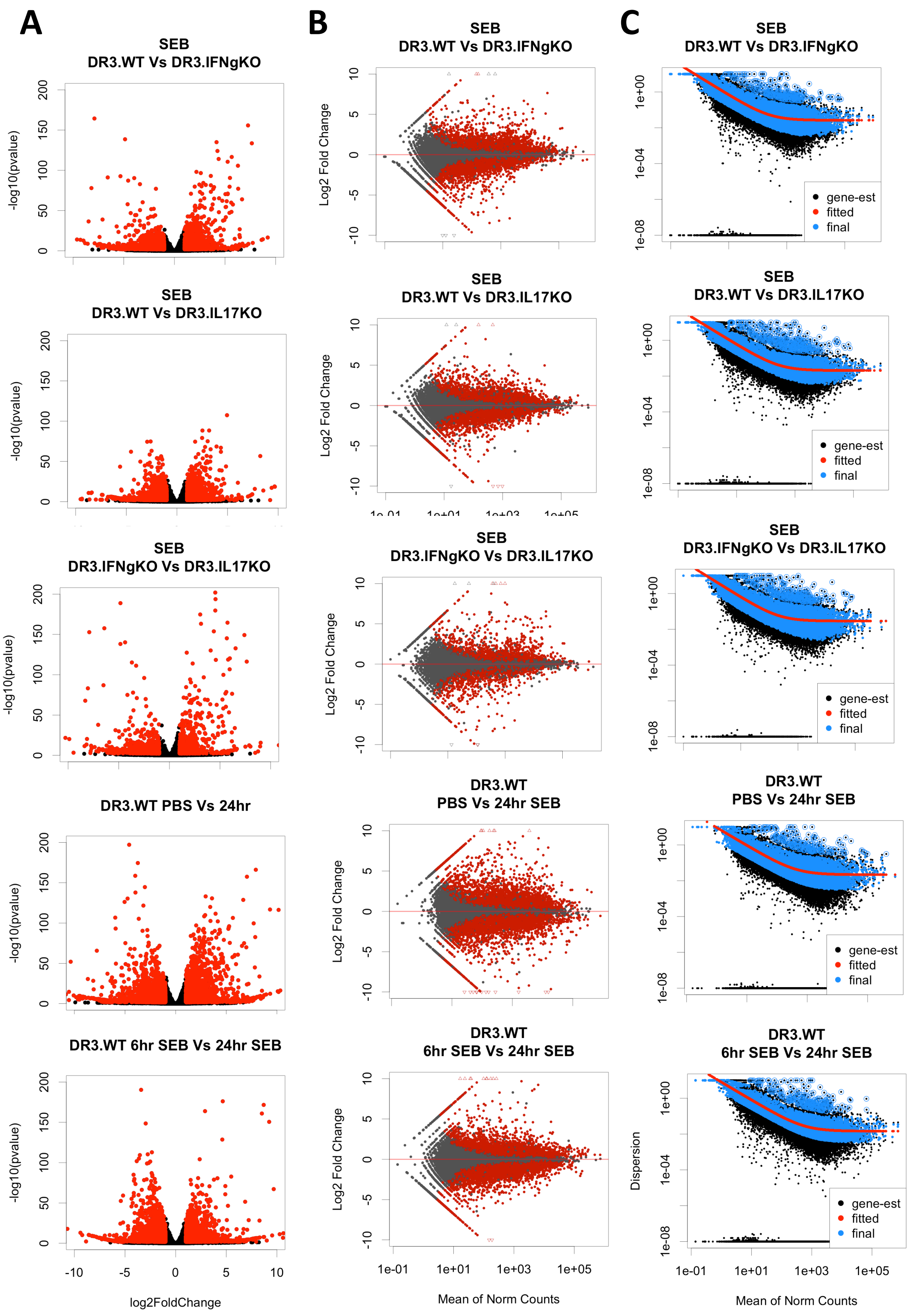

Supp. Figure 1.

Supp. Figure 1. Analytics on differential expression. (A) Volcano plot of Log2 Fold Change against -Log10 Padj. Genes where Padj < 0.05 and | Log2 Fold Change | [illegible] 1 are colored in red. (B) MA plot for Mean of Norm counts (Across both conditions) versus Log2 Fold Change between two conditions. Genes where Padj < 0.05 and | Log2 Fold Change | [illegible] 1 are colored in red. (C) Dispersion plot for each specific pair-wise comparison using the mean of normalized counts versus dispersion estimate. Each genes dispersion is independently determined via maximum likelihood estimate (black dot). A red curve is fitted to model the relationship between the dispersion and mean values to generate the dispersion parameter. The final dispersion for each gene based on shrinkage estimation using the dispersion parameter.

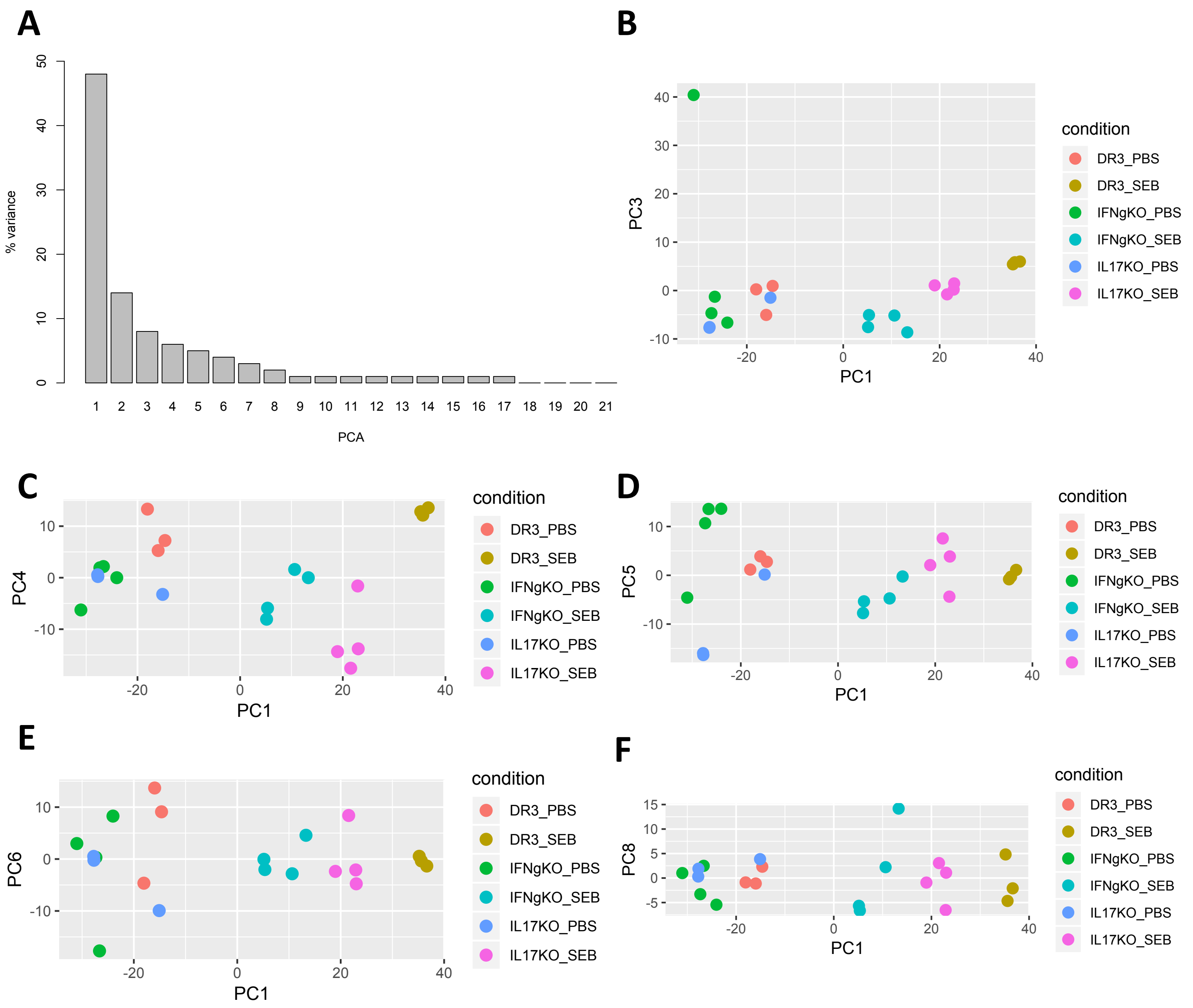

Supp. Figure 2. Analysis of derived principal components associated with global analysis of normalized counts. (A) Percentage of variance accounted for by each component. (B-F) Plotting PC3-8 against PC1.

A DEGs (Increasing) in Response to SEB by DR3.WT

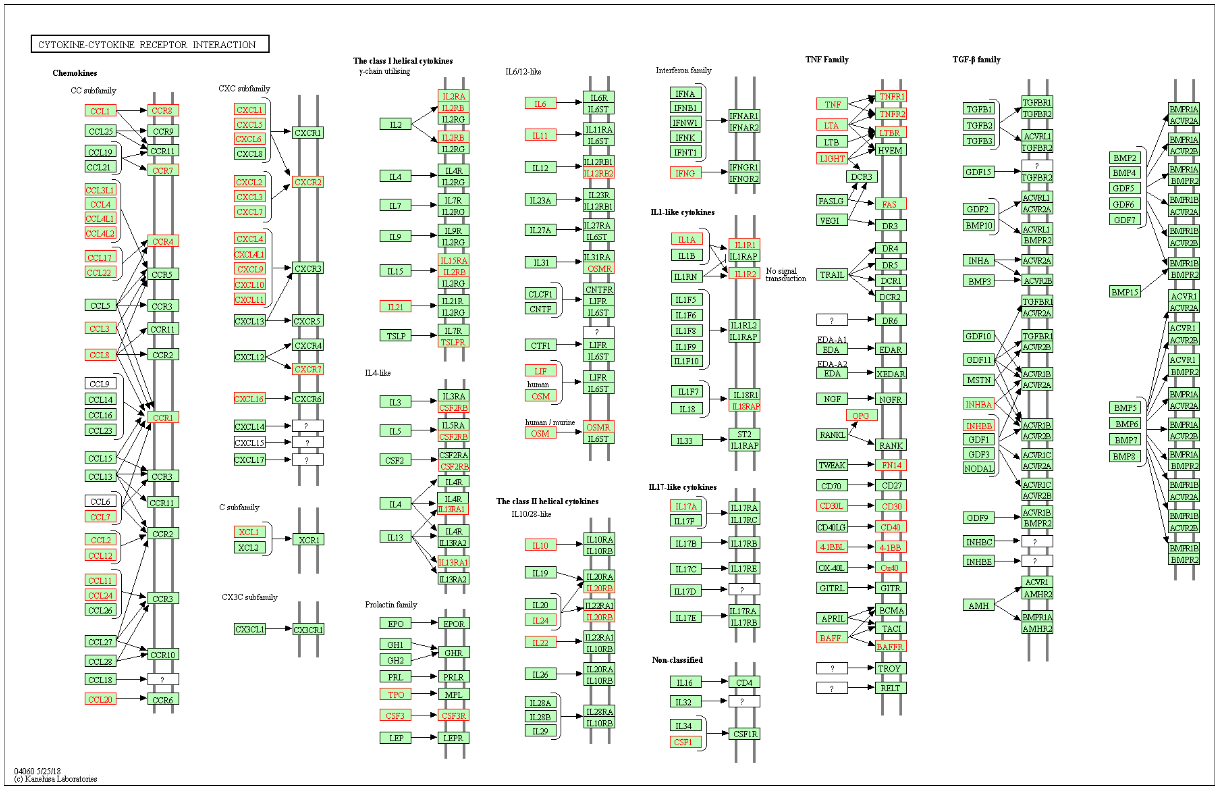

B DEGs (Increasing) in Response to SEB by DR3.WT

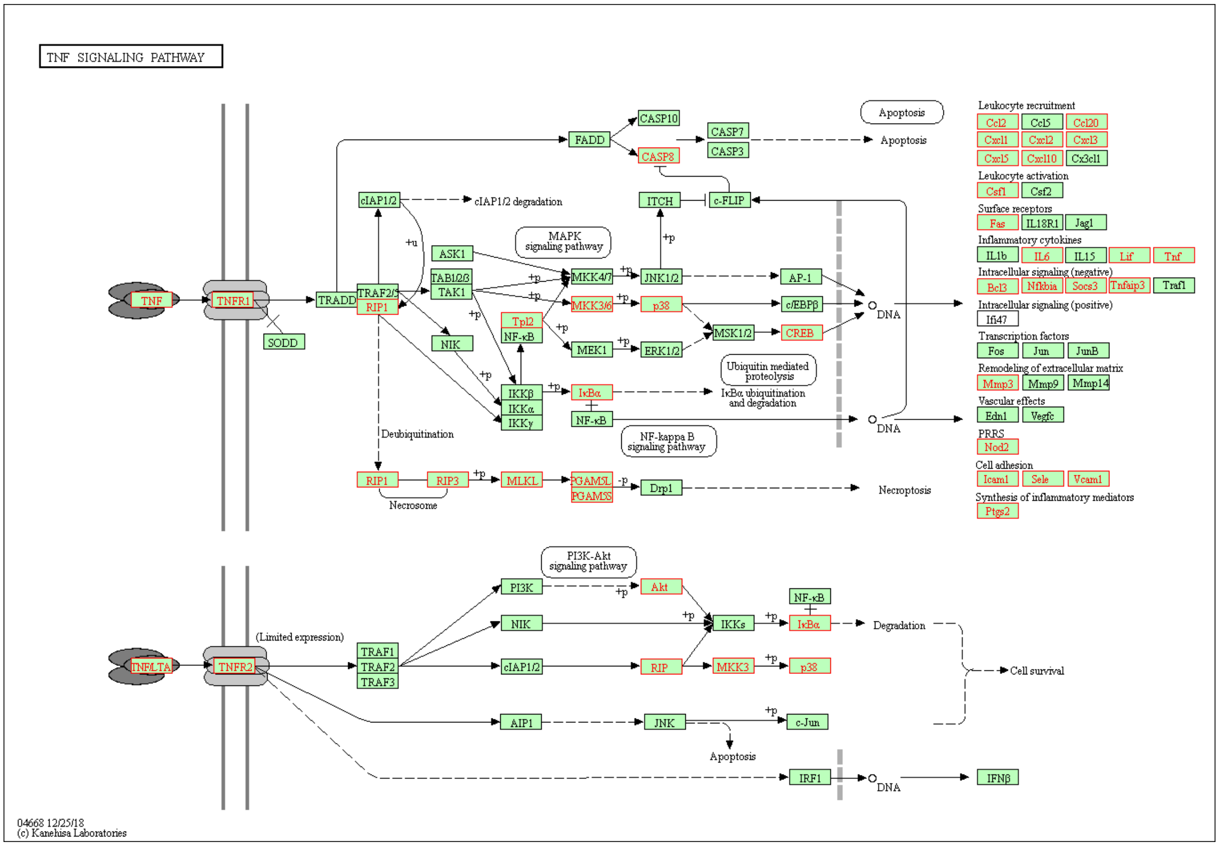

Supp. Figure 3

C DEGs (Increasing) in Response to SEB by DR3.WT

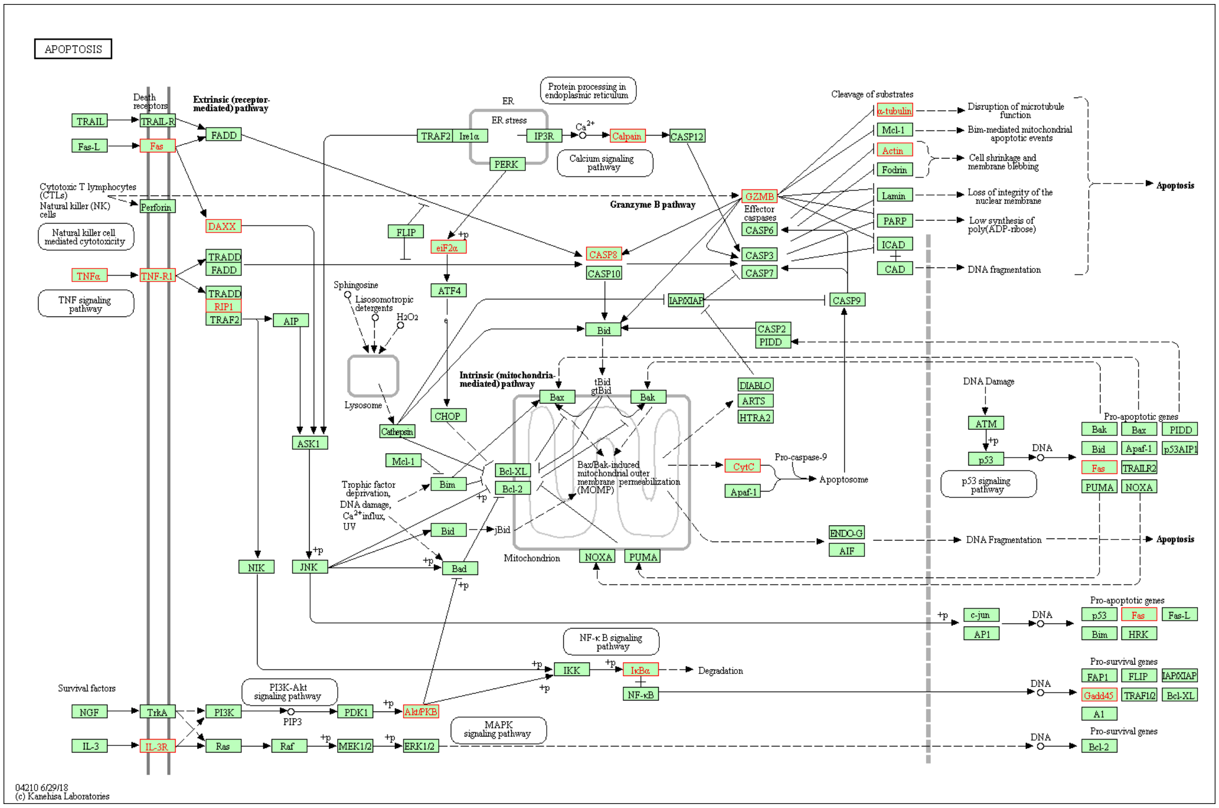

D DEGs (Increasing) in Response to SEB by DR3.WT

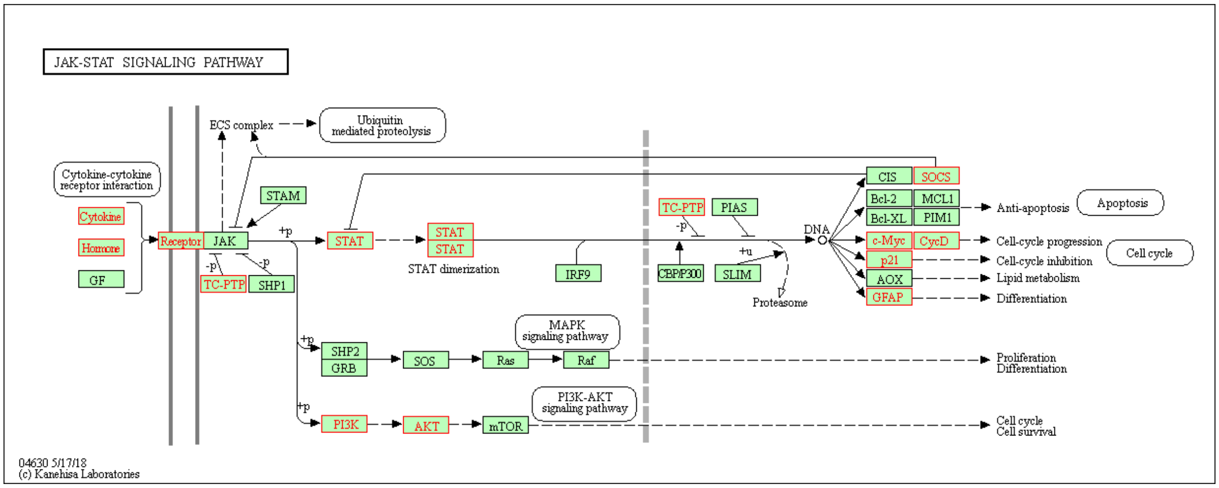

Supp. Figure 3

E **DEGs (Increasing) in Response to SEB by DR3.WT**

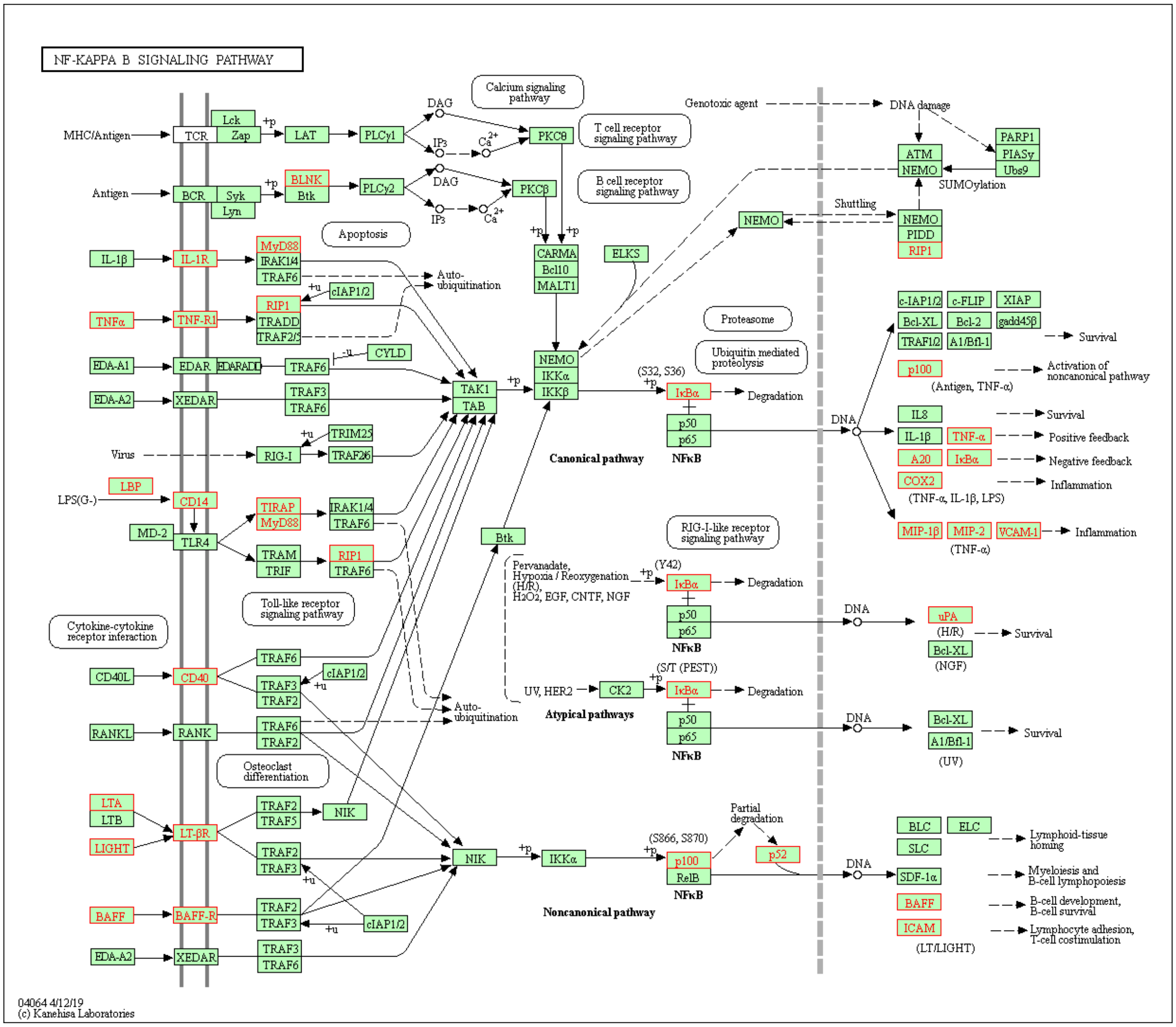

F **DEGs (Decreasing) in Response to SEB by DR3.WT**

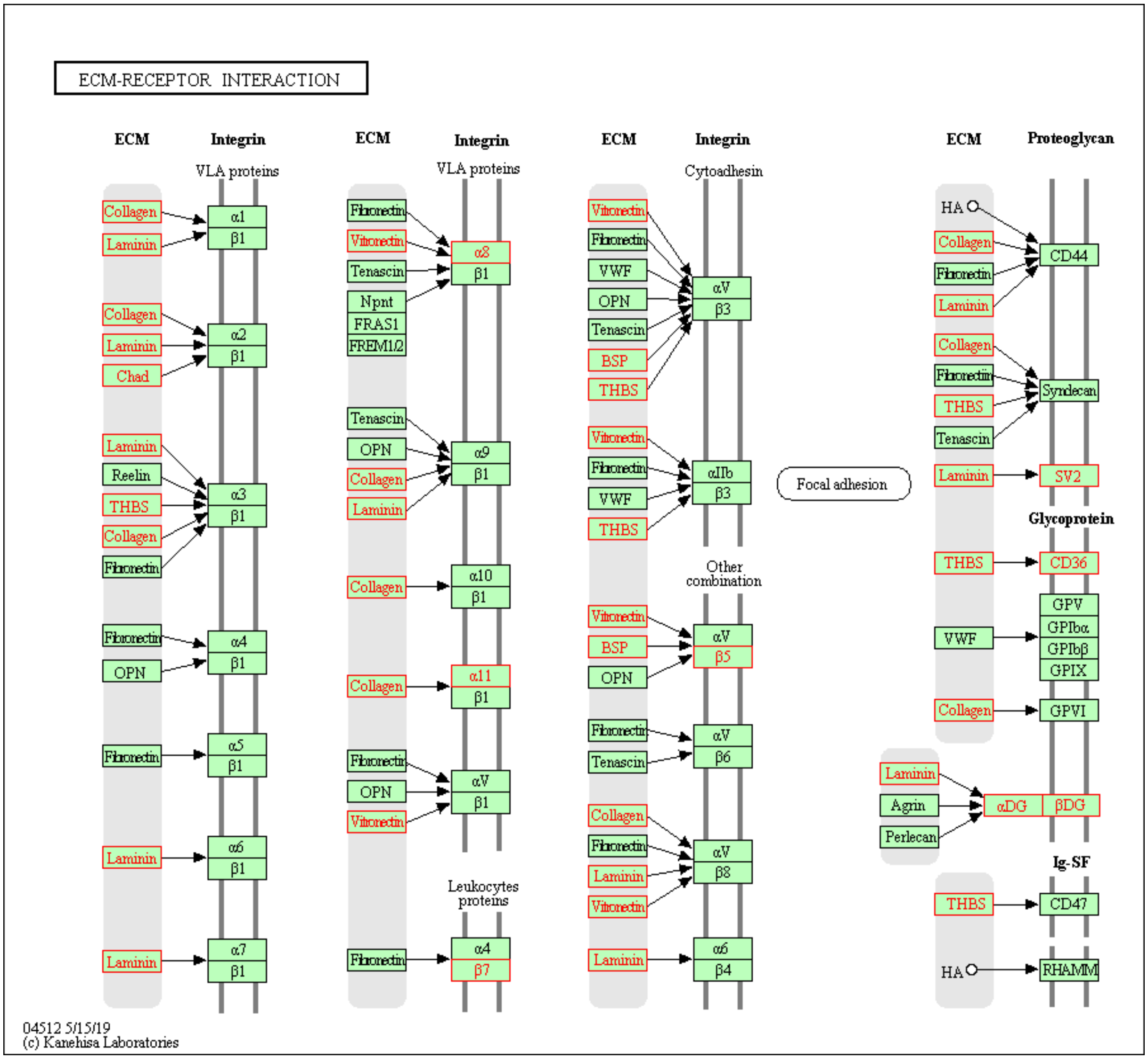

Supp. Figure 3. <u>Subset of Pathways Enriched by DR3.WT DEGs in response to SEB. Enriched Pathways were visualized using the KEGG pathway maps</u>. Pathways enriched by DEGs increasing in expression include: (A) Cytokine-cytokine receptor interaction, (B) TNF signaling pathway, (C) Apoptosis, (D) Jak-Stat Signaling Pathway, and (E) NFκB signaling pathway. Pathways enriched by DEGs decreasing in expression include: (F) ECM-Receptor interaction.

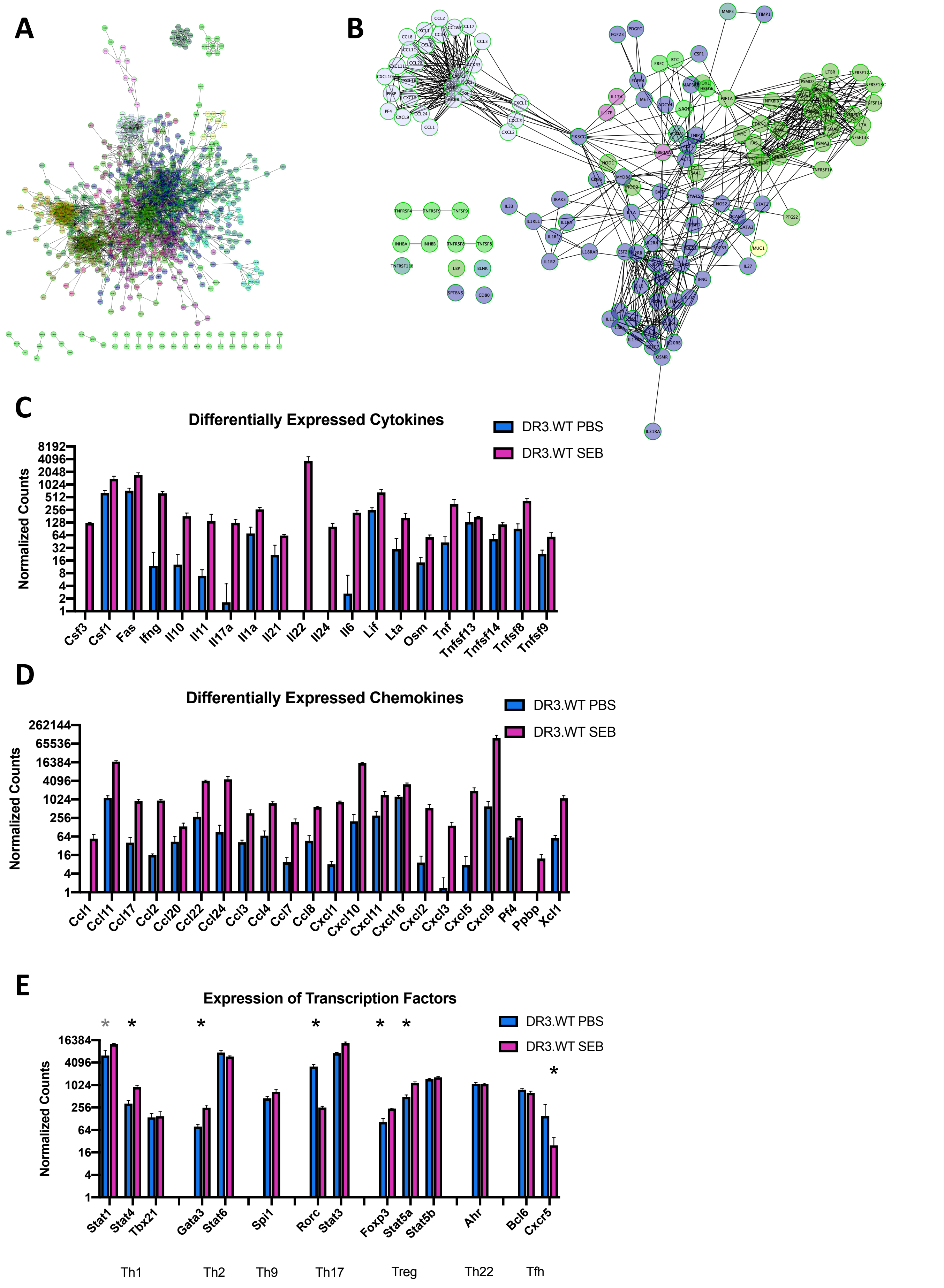

Supp. Figure 4

Supp. Figure 4. Identification of differentially expressed cytokines and chemokines in response to SAg-induced CRS in DR3.WT mice. (A) Differentially expressed genes overlaid onto the reactome protein-protein interaction map, sub networks are color-coded based on enriched pathways. (B) Protein-protein Interaction network of cytokine chemokines and their receptors that were differentially expressed in an increasing manner in response to SEB. Normalized expression of differentially expressed (C) cytokines and (D) chemokines in response to SEB in DR3.WT (padj <0.05, log2fold change >1). (E) Expression of known transcription factor regulators associated with immune signaling Asterisk indicates differential expression (padj <0.05, log2fold change >1).

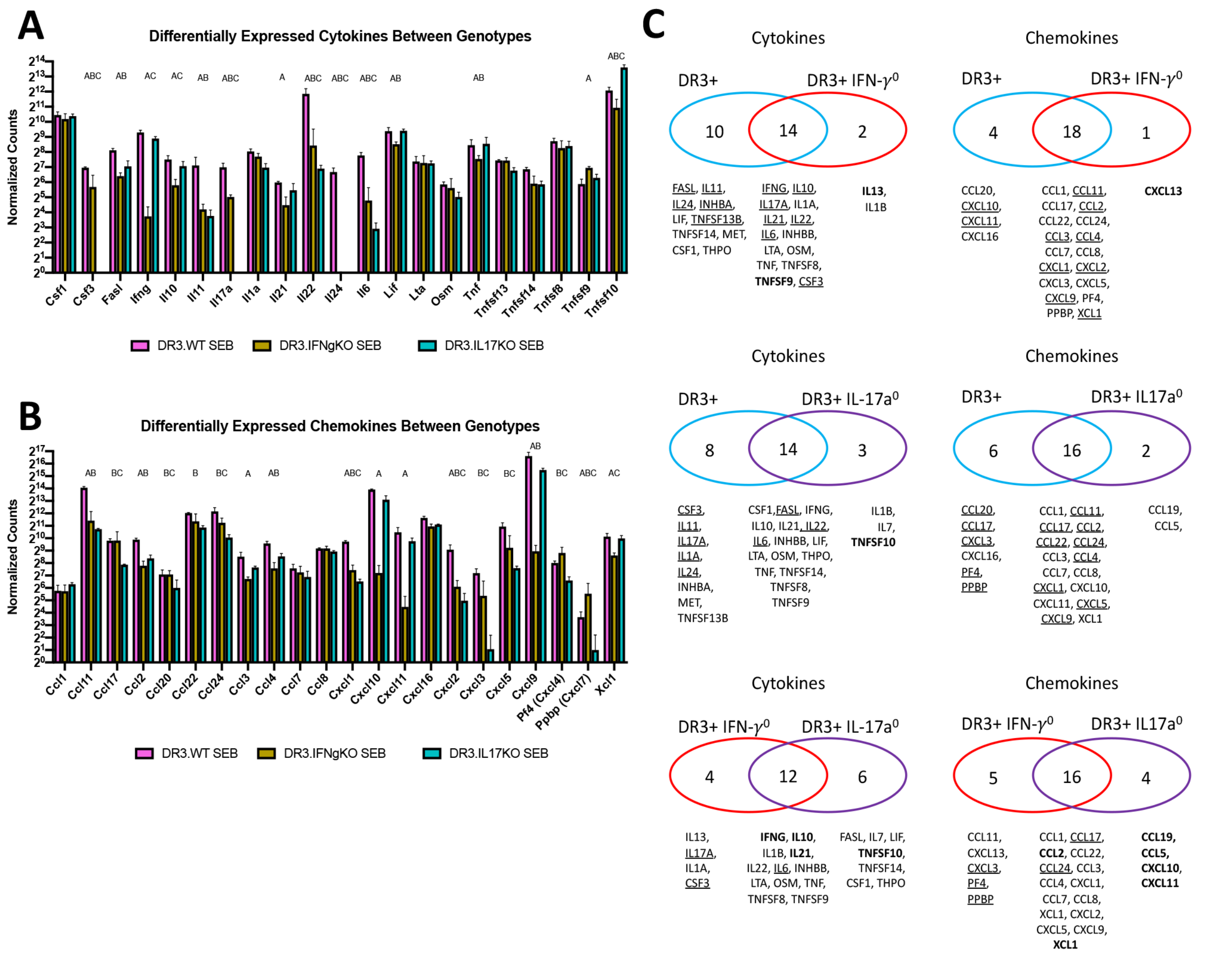

Supp. Figure 5. Comparison of cytokine and chemokine genes differentially expressed across all genotypes during SA-induced CRS. (A and B) Normalized expression values for differentially expressed cytokines and chemokine genes between DR3.WT, DR3.IFN-γo, and DR3.IL17o mice treated with SEB. (C) Overlap of differentially expressed cytokines and chemokines in response to SEB between each genotype. Underlined values were also considered differentially expressed between the two genotypes in an increasing manner for the group on the left, while bold values are for the right. For A, B and C above the bars, ‘A’ indicates DE between DR3.WT and DR3.IFN-γ^{o}. ‘B’ indicates DE between DR3.WT and DR3.IL-17°. ‘C ‘indicates DE between DR3.IFN-γ^{o} and DR3.IL-17° mice.

A **Enriched DR3.WT Versus DR3.IFNgKO with SEB**

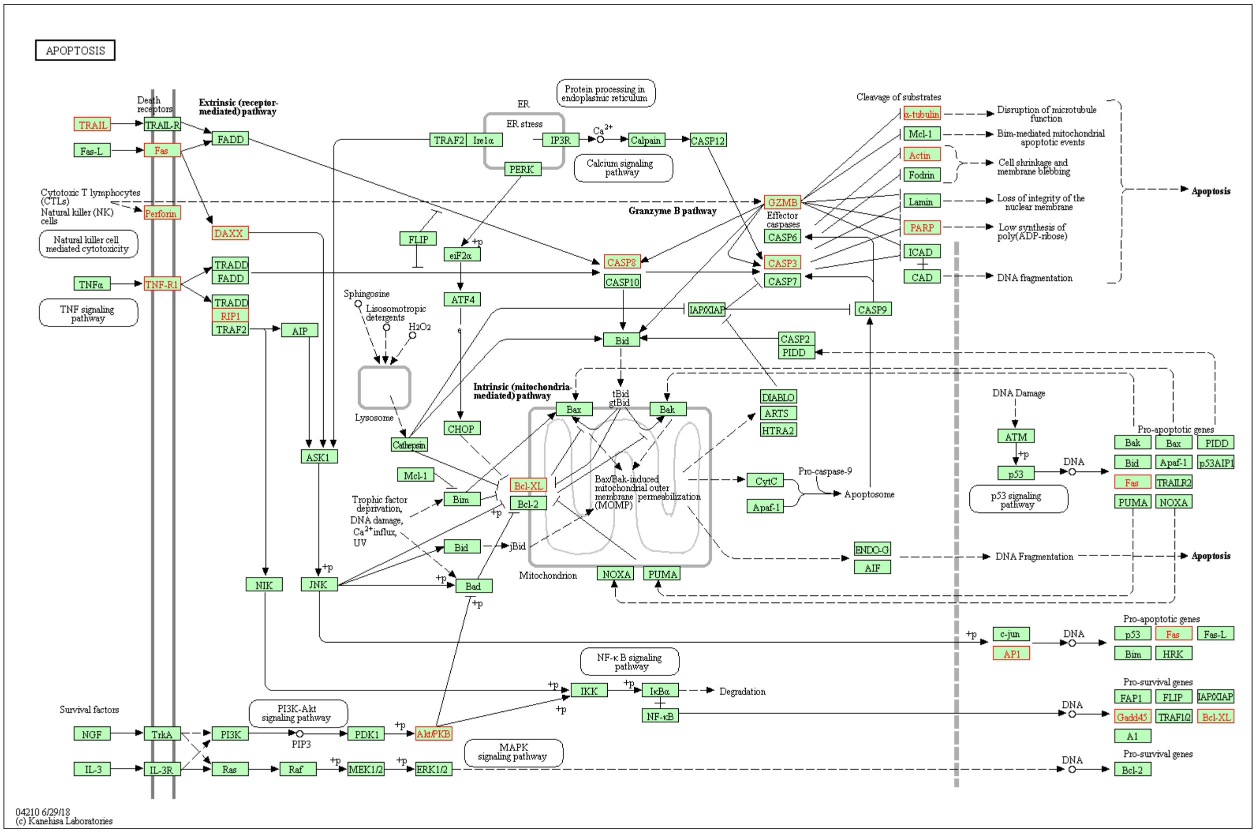

B **Enriched DR3.IL17KO Versus DR3.IFNgKO with SEB**

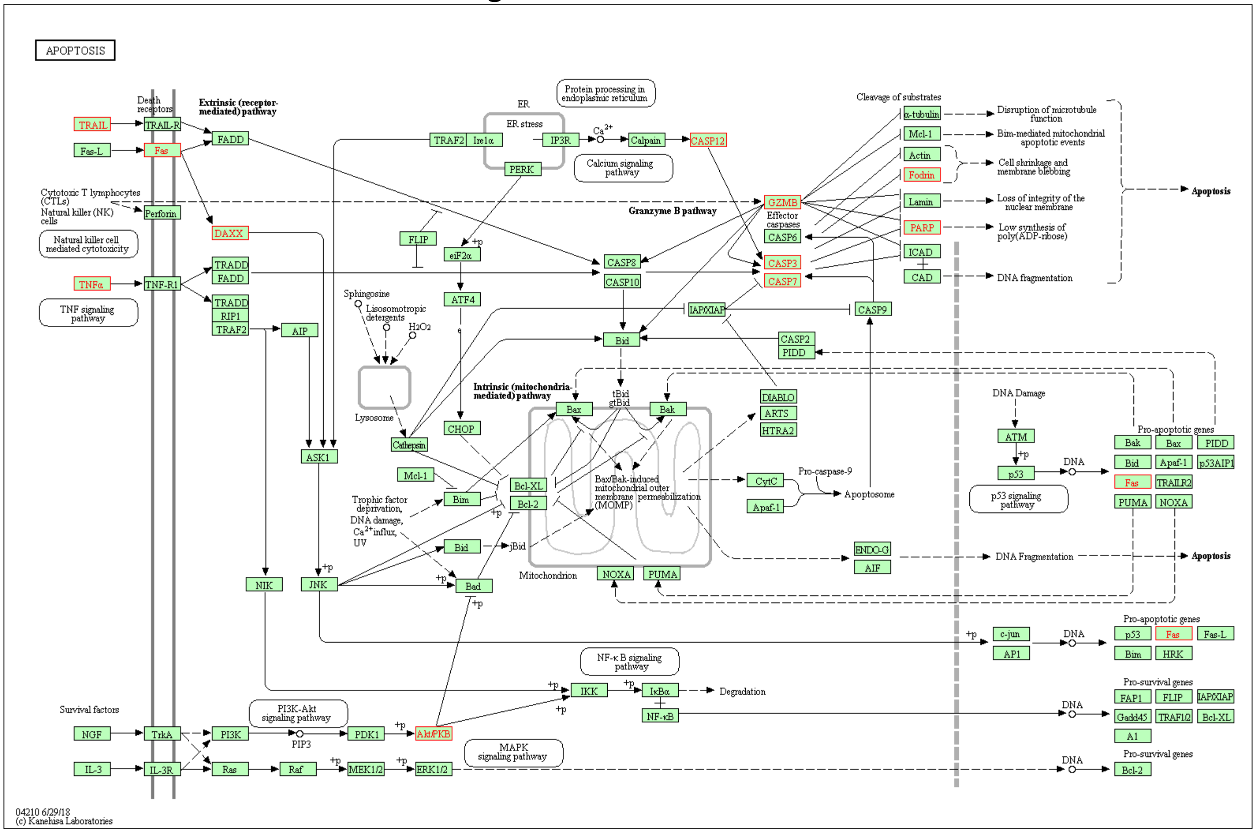

Supp. Figure 6. Subset of Pathways Enriched between DR3.WT, DR3.IFN-γ° and DR3.IL-17° mice treated with SEB.

Supp. Fig 7

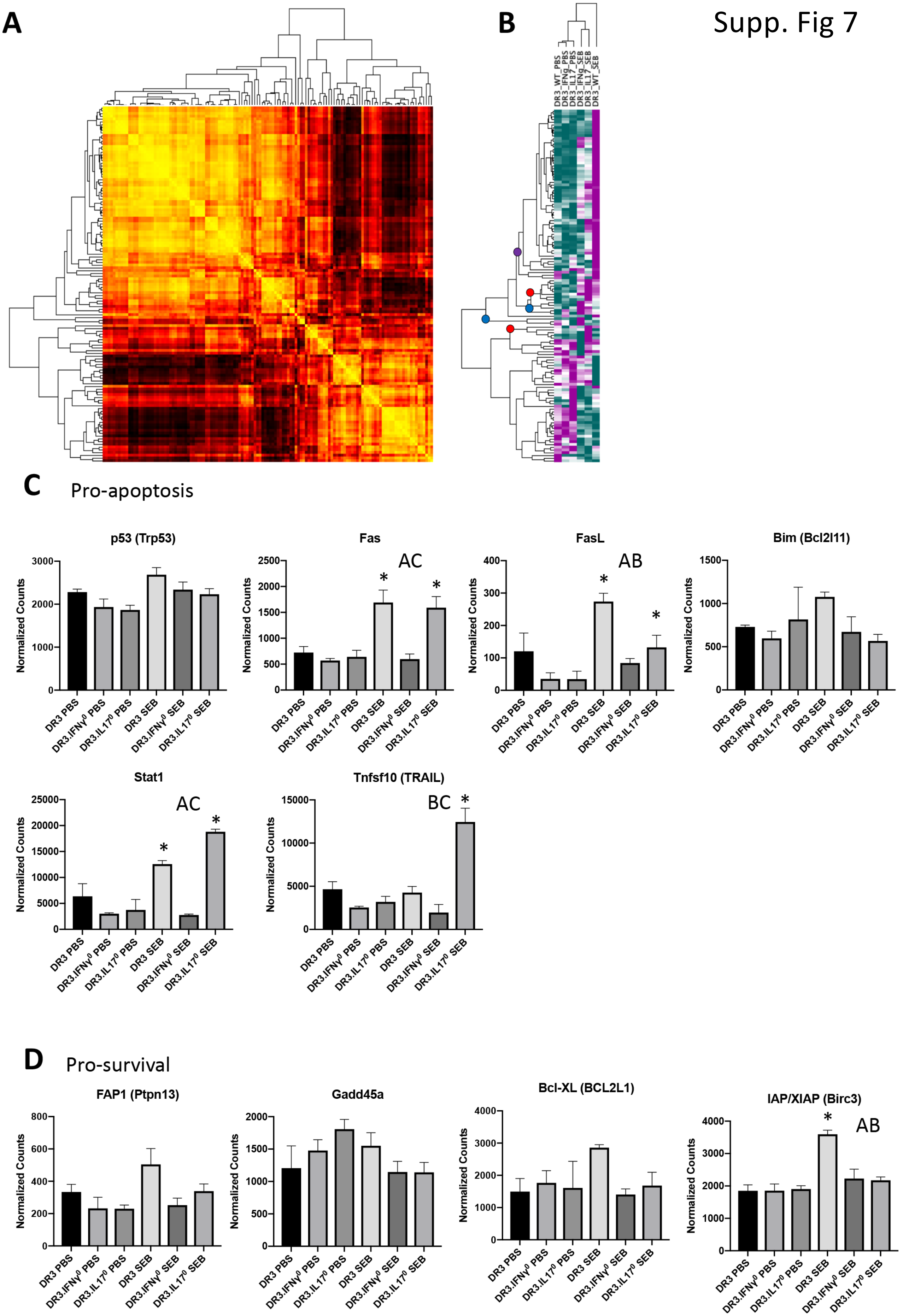
A
B
DR3_WT_PBS
DR3_IFNg_PBS
DR3_IL17_PBS
DR3_IFNg_SEB
DR3_IL17_SEB
DR3_WT_SEB
C
Pro-apoptosis
p53 (Trp53)
Fas
AC
FasL
AB
Bim (Bcl2l11)
Stat1
AC
Tnfsf10 (TRAIL)
BC
Normalized Counts
DR3 PBS
DR3.IFNγ0 PBS
DR3.IL170 PBS
DR3 SEB
DR3.IFNγ0 SEB
DR3.IL170 SEB
D
Pro-survival
FAP1 (Ptpn13)
Gadd45a
Bcl-XL (BCL2L1)
IAP/XIAP (Birc3)
AB

E

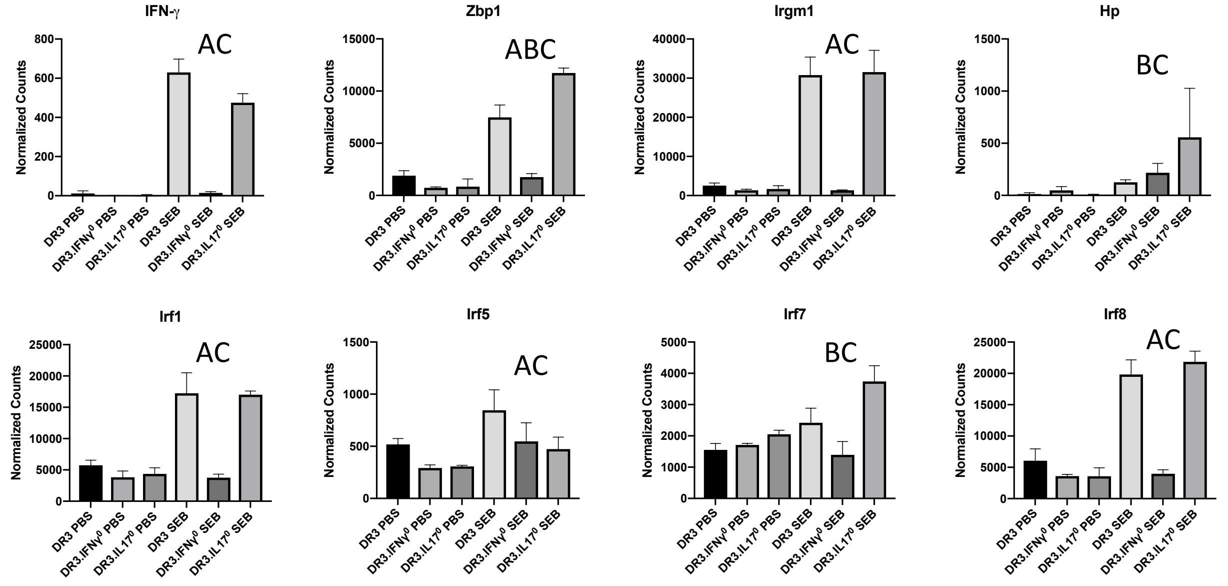

Supp. Figure 7. Identification of differential expressed genes associated with apoptosis in SA-induced CRS. (A) Hierarchical clustering of genes included in the apoptosis pathway (B) Relative expression of these genes. Circles colored nodes indicates a subset of genes used to overlay on the larger KEGG reaction map (Not shown). Purple, blue, and red indicate genes with the highest expression in DR3.WT, DR3.IFN-γo and DR3.IL17o mice treated with SEB, respectively. Normalized expression for selected pro-apoptotic (C) and pro-survival genes (D) across all genotypes and conditions. (E) Normalized expression for IFN-γ and IFN-γ inducible genes. Differential expression (padj <0.05, log2fold D > 1) between genotypes and treatment is indicated by symbol. Star indicates DE between treatment groups (PBS versus SEB); 'A' indicates DE between DR3.WT and DR3.IFN-γo. 'B' indicates DE between DR3.WT and DR3.IL 17o. 'C 'indicates DE between DR3.IFN-γo and DR3.IL-17o mice.

Supp. Fig 8

A Differentially expressed genes enriched in DR3.IFN-γ^{o} in comparison to DR3.WT

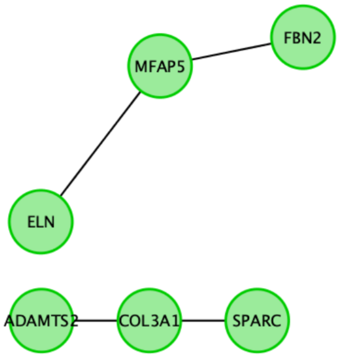

elastic fibers (ELN, FBN2)
fibrillar collagen (COL3A1)
connective tissue microfibrils (FBN2, MFAP5)
matrix-associated proteins (ADAMTS2, SPARC)

B Clustering of genes associated with ECM-Receptor Interactions

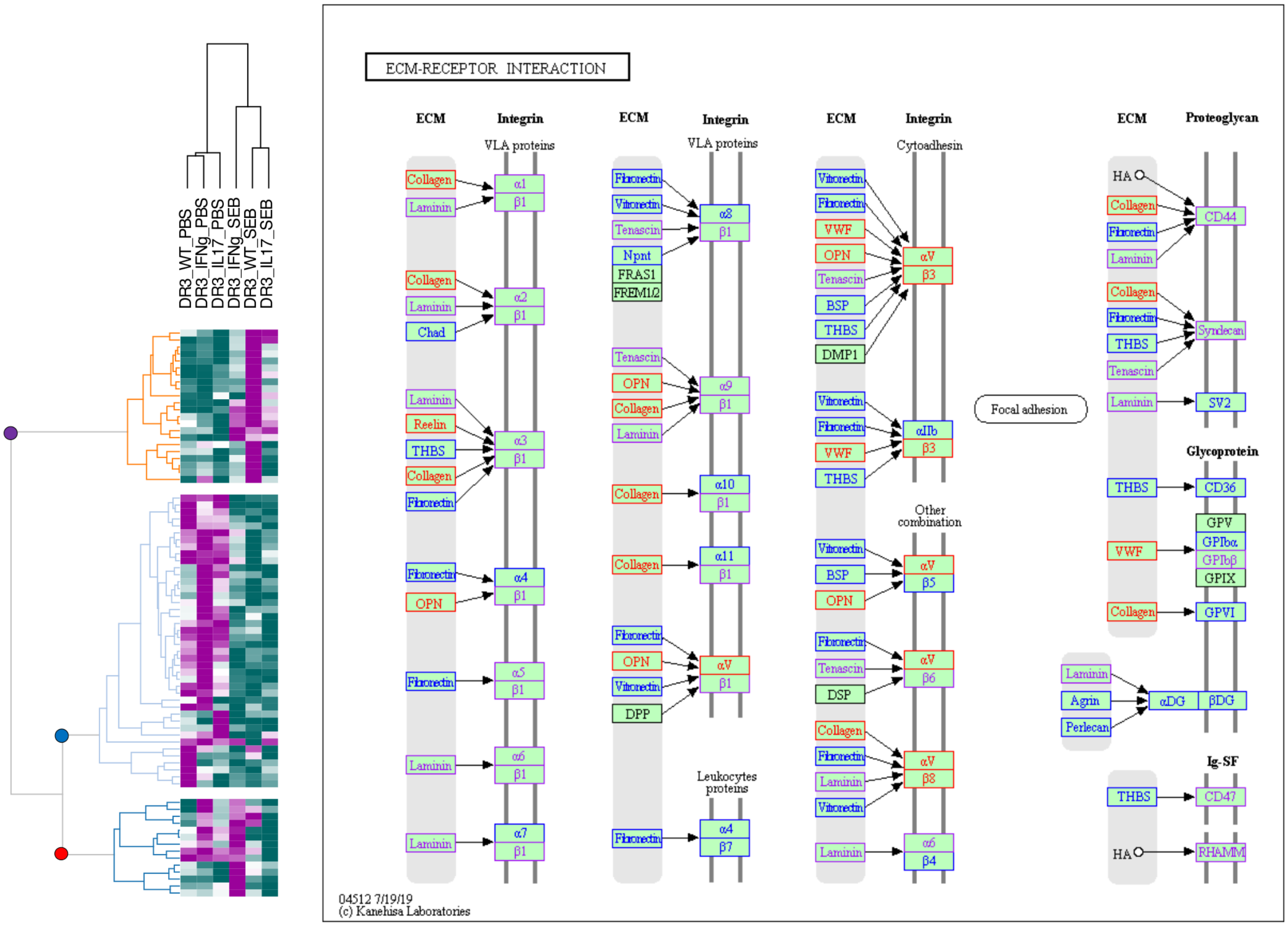

Supp. Figure 8. Differentially expressed genes and clustering of genes associated with ECM-receptor interactions in resting state and during SA-induced CRS. (A) Differentially expressed genes enriched in DR3.IFN-γ^{o} in comparison to DR3.WT treated with PBS. (B) Hierarchical clustering of genes expression associated with ECM-Receptor Interactions. Expression is shown in a row relative manner: Green lowest expression, Purple is highest expression. Red dot/text indicates cluster where highest expression was in DR3.IFN-γ^{o} treated with SEB. Blue dot/test indicates highest expression in control PBS treated mice across all genotypes. Purple dot/text indicates highest expression in DR3.WT mice treated with SEB.

Supp. Figure 9

A

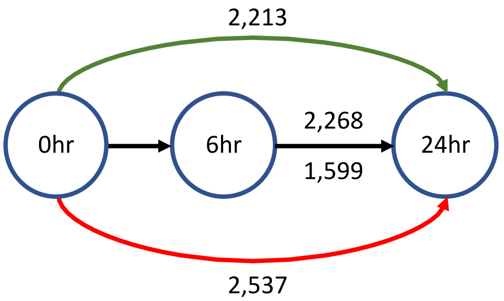

B

Expression Groupings	DEGs	Nodes	Edges	E/N Ratio	Pathways Enriched (FDR <0.005)
A	113	17	15	0.88	13
B	569	114	164	1.44	40
C	1031	234	318	1.36	17
D	138	5	9	1.80	10
E	1,632	593	2,379	4.01	42
F	1,127	290	631	2.18	34
G	892	155	280	1.81	3
H	1,950	345	624	1.81	45

C

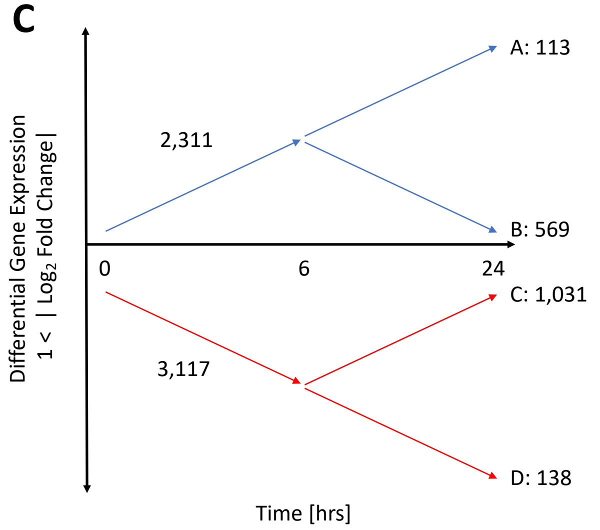

D

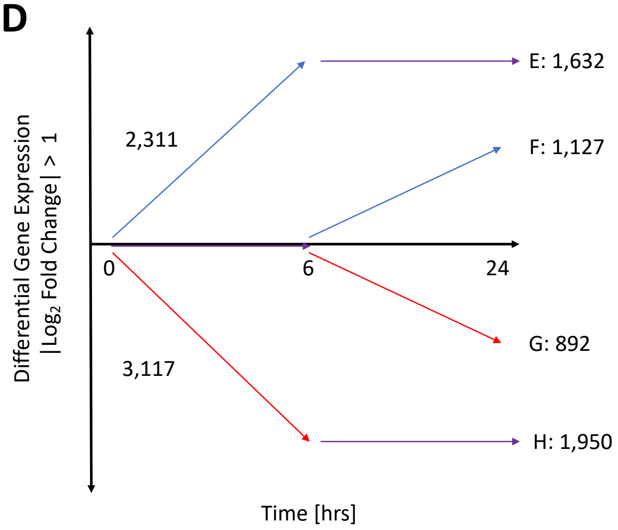

E Group G enrichment

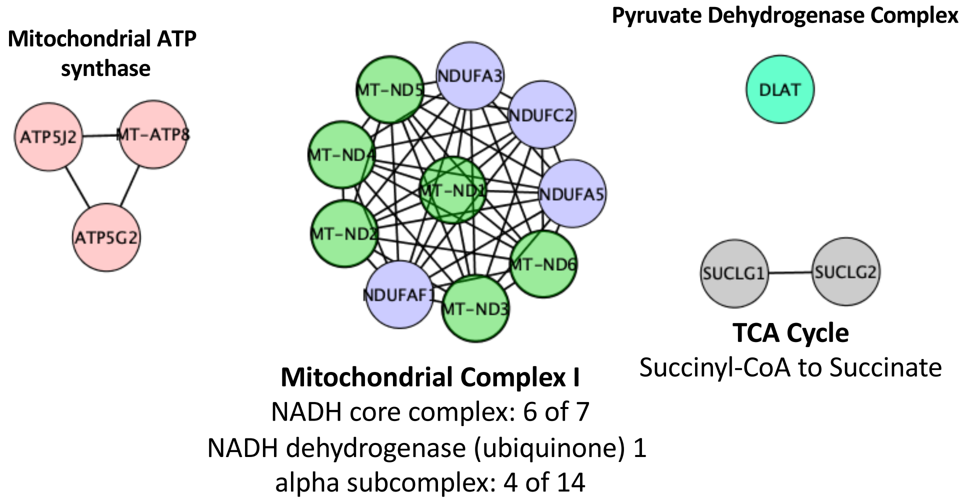

Supp. Figure 9. Differential gene expression in response to SEB at 24 hours. Genes were first filtered out of the data set when a given gene had an expression value below 10 across all compared sample replicates. A) Number of differentially expressed genes (log2fold D >1 , padj < 0.05) in comparison to DR3.WT naïve and 6 hours post SEB. B) Summary of differential expression patterns between DR3.WT nave (0 hr), 6 hours post SEB, and 24 hours post SEB. (CD) Differential expression patterns and the associated number of genes for each pattern. (E) Enriched Clusters from protein-protein interaction and group G expression members.

Supplementary Table 1: Summary of RNA seq Read Quality and Alignment. Number and percentage of paired end reads was determined after trimming poor quality bases and filtering out unpaired reads. Alignment of paired end reads to the mouse reference genome indicated the vast majority of reads aligned once to a specific loci, while a minority (7-10%) aligned to multiple loci.

Condition	Sampe ID	Paired End Reads	% of PE Reads	aligned concordantly exactly 1 time		aligned concordantly >1 times		aligned concordantly 0 times		Overall Alignment Rate
DR3+ with PBS	S10	24,060,169	100%	21,435,812	89.09%	1,969,666	8.19%	654,691	2.72%	98.79%
DR3+ with PBS	S11	26,047,217	100%	23,021,974	88.39%	2,348,624	9.02%	676,619	2.60%	98.65%
DR3+ with PBS	S12	24,869,661	100%	21,661,418	87.10%	2,563,479	10.31%	644,764	2.59%	98.80%
DR3+ IFNg KO with PBS	T11	36,169,543	100%	32,701,066	90.41%	2,619,387	7.24%	849,090	2.35%	98.96%
DR3+ IFNg KO with PBS	S13	27,034,587	100%	23,934,109	88.53%	2,328,007	8.61%	772,471	2.86%	98.96%
DR3+ IFNg KO with PBS	S14	22,390,719	100%	19,639,111	87.71%	1,999,030	8.93%	752,578	3.36%	98.73%
DR3+ IFNg KO with PBS	S15	25,251,785	100%	22,180,569	87.84%	2,168,348	8.59%	902,868	3.58%	98.86%
DR3+ IL17a KO with PBS	S17	24,004,531	100%	21,299,205	88.73%	2,044,432	8.52%	660,894	2.75%	98.76%
DR3+ IL17a KO with PBS	S18	23,219,119	100%	20,574,569	88.61%	1,896,428	8.17%	748,122	3.22%	98.50%
DR3+ IL17a KO with PBS	T7	37,150,043	100%	32,575,225	87.69%	3,365,212	9.06%	1,209,606	3.26%	99.00%
DR3+ with SEB	S1	22,260,630	100%	19,710,099	88.54%	2,015,226	9.05%	535,305	2.40%	98.69%
DR3+ with SEB	S2	22,652,969	100%	19,676,557	86.86%	2,400,003	10.59%	576,409	2.54%	98.77%
DR3+ with SEB	S3	22,367,487	100%	19,283,847	86.21%	2,353,148	10.52%	730,492	3.27%	98.77%
DR3+ IFNg KO with SEB	S4	21,724,956	100%	18,797,103	86.52%	2,192,221	10.09%	735,632	3.39%	98.85%
DR3+ IFNg KO with SEB	S6	23,069,460	100%	20,147,050	87.33%	2,030,000	8.80%	892,410	3.87%	98.69%
DR3+ IFNg KO with SEB	T12	32,758,560	100%	28,767,175	87.82%	2,518,900	7.69%	1,472,485	4.49%	98.82%
DR3+ IFNg KO with SEB	T13	34,964,927	100%	30,600,175	87.52%	2,797,611	8.00%	1,567,141	4.48%	98.84%
DR3+ IL17a KO with SEB	S8	22,185,487	100%	19,359,570	87.26%	1,992,618	8.98%	833,299	3.76%	98.96%
DR3+ IL17a KO with SEB	T4	36,659,308	100%	32,087,391	87.53%	3,636,582	9.92%	935,335	2.55%	99.09%
DR3+ IL17a KO with SEB	T5	36,382,740	100%	31,473,662	86.51%	3,725,513	10.24%	1,183,565	3.25%	99.05%
DR3+ IL17a KO with SEB	T6	33,341,293	100%	28,871,783	86.59%	3,464,349	10.39%	1,005,161	3.01%	98.98%
DR3+ with SEB (24hr)	T15	37,006,079	100%	33,068,153	89.36%	2,869,284	7.75%	1,068,642	2.89%	98.92%
DR3+ with SEB (24hr)	T16	34,520,137	100%	31,013,686	89.84%	2,643,327	7.66%	863,124	2.50%	98.97%

Supplementary Table 2: Summary of Differentially Expressed Genes. The number of differentially expressed genes was determined for each type of listed comparison, where the padj < 0.05. Differentially expressed genes were further filtered based on criteria that the absolute value of the log2 fold change was greater than 1. The percentage reduction is in reference to the decrease in differentially expressed genes when both criteria are required rather then just the padj < 0.05.

		Number of DEGs					
		Padj < 0.05		Filtered $\lvert log_2 Change \rvert$ >1		% Reduction	
**Genotype**	**Condition**	**Increase**	**Decrease**	**Increase**	**Decrease**	**Increase**	**Decrease**
DR3.WT	PBS Vs SEB	5,100	5,258	2,311	3,117	54.69	40.72
DR3.IFNgKO	PBS Vs SEB	3,511	3,809	1,116	1,624	68.21	57.36
DR3.IL17KO	PBS Vs SEB	4,042	3,967	1,485	1,667	63.26	57.98
DR3.WT	PBS Vs SEB(24hr)	4,585	4,587	2,213	2,537	47.27	54.31
DR3.WT	SEB(6hr) Vs SEB(24hr)	4,329	4,179	2,268	1,599	51.39	37.26
Enriched 1 Vs Enriched 2	Condition	Enriched 1	Enriched 2	Enriched 1	Enriched 2	Enriched 1	Enriched 2
DR3.WT Vs DR3.IFNgKO	PBS	788	528	410	267	47.97	49.43
DR3.WT Vs DR3.IL17KO	PBS	644	453	356	137	44.72	69.76
DR3.IFNgKO Vs DR3.IL17KO	PBS	535	624	229	383	57.20	38.62
DR3.WT Vs DR3.IFNgKO	SEB	3,434	3,663	1,137	1,768	66.89	51.73
DR3.WT Vs DR3.IL17KO	SEB	3,283	3,363	1,054	1,374	67.90	59.14
DR3.IFNgKO Vs DR3.IL17KO	SEB	1,620	1,521	694	661	57.16	56.54
					**Average:**	**56.97**	**52.07**

Supplementary Table 3: Summary of Pathway Enrichment. Differentially expressed genes (DEGs) were overlaid onto the reactome protein-protein interaction network, and a pathway enrichment analysis was conducted for Reactome, KEGG, Panther DB, and NCI pathway libraries. DEGs: Differentially Expressed genes. Nodes: Identified proteins in the Reactome protein-protein interaction network. Edges: Connections between proteins known to have physical interactions. Edge/ Node Ratio: The total number of edges divided by nodes. Node Discovery: The number of identified nodes from the list of DEGs. Pathways Enriched: The number of pathways enriched that have a p < 0.01, FDR.

Genotype	Condition	Enrichment	DEGs	Nodes	Edges	Edge/Node Ratio	Node Discovery	Pathways Enriched
DR3.WT	PBS Vs SEB	Increase	2,311	859	4020	4.680	37.170%	58
		Decrease	3,118	741	1907	2.574	23.765%	74
DR3.IFNgKO	PBS Vs SEB	Increase	1,116	289	651	2.253	25.896%	65
		Decrease	1,624	318	483	1.519	19.581%	34
DR3.IL17KO	PBS Vs SEB	Increase	1,485	465	1431	3.077	31.313%	68
		Decrease	1,667	329	534	1.623	19.736%	32
DR3.WT Vs DR3.IFNgKO	PBS	DR3.WT	410	75	174	2.320	18.293%	44
		DR3.IFNgKO	267	6	4	0.667	2.247%	3
DR3.WT Vs DR3.IL17KO	PBS	DR3.WT	356	86	192	2.233	24.157%	45
		DR3.IL17KO	137	4	2	0.500	2.920%	2
DR3.IFNgKO Vs DR3.IL17KO	PBS	DR3.IFNgKO	229	13	9	0.692	5.677%	12
		DR3.IL17KO	383	5	3	0.600	1.305%	2
DR3.WT Vs DR3.IFNgKO	SEB	DR3.WT	1,137	304	767	2.523	26.737%	93
		DR3.IFNgKO	1,768	291	467	1.605	16.459%	17
DR3.WT Vs DR3.IL17KO	SEB	DR3.WT	1,054	233	441	1.893	22.106%	59
		DR3.IL17KO	1,374	212	282	1.330	15.429%	9
DR3.IFNgKO Vs DR3.IL17KO	SEB	DR3.IFNgKO	694	138	275	1.993	19.885%	38
		DR3.IL17KO	661	55	67	1.218	8.321%	16
DR3.WT	PBS Vs SEB (24hr)	Increase	2,213	781	2,956	3.785	35.29%	118
		Decrease	2,537	517	954	1.845	20.38%	35
DR3.WT	SEB (6hr) Vs SEB (24hr)	Increase	2,268	708	2,055	2.902	31.22%	45
		Decrease	1,599	382	804	2.105	23.89%	23
